# The Identification of a Key Regulator of Mitochondrial Metabolism, the LRPPRC Protein, as a Novel Therapeutic Target in SDHA-Overexpressing Ovarian Tumors

**DOI:** 10.3390/cancers17121942

**Published:** 2025-06-11

**Authors:** Anna Szulta, Lin Wang, Ameera Hasan, Michael Kinter, Atul Pranay, Jie Zhu, Kenneth M. Humphries, Brooke Loveland, Timothy M. Griffin, Magdalena Bieniasz

**Affiliations:** 1 Aging and Metabolism Research Program, Oklahoma Medical Research Foundation, Oklahoma City, OK 73104, USA; anna-szulta@omrf.org (A.S.); lin-wang@omrf.org (L.W.); ameera-hasan@omrf.org (A.H.); mike-kinter@omrf.org (M.K.); atul-pranay@omrf.org (A.P.); kenneth-humphries@omrf.org (K.M.H.); brooke-loveland@omrf.org (B.L.); tim-griffin@omrf.org (T.M.G.); 2 Cardiovascular Biology Research Program, Oklahoma Medical Research Foundation, Oklahoma City, OK 73104, USA; jie-zhu@omrf.org

**Keywords:** ovarian cancer, succinate dehydrogenase, SDHA, LRPPRC, OXPHOS, high-grade serous ovarian cancer, shikonin, metabolism, patient-derived xenograft

## Abstract

**Background:** Ovarian cancer is the deadliest of all gynecologic malignancies due to limited therapeutic options. Our data show that the tumor-specific metabolism of ovarian cancer could be effectively targetable, which highlights a path for new anti-cancer therapies. **Methods and Results:** Our work shows that the upregulation of mitochondrial enzyme SDHA is particularly prevalent in ovarian carcinoma. SDHA overexpression significantly induced orthotopic ovarian tumor growth, reducing mouse survival. We showed that SDHA-overexpressing tumors depend on glutaminolysis and increased activity of the tricarboxylic acid (TCA) cycle coupled with mitochondrial oxidative phosphorylation (OXPHOS), which are essential for high-energy metabolism and cell survival. We identified a distinctive vulnerability of SDHA-overexpressing tumors to agents targeting regulators of the OXPHOS pathway, particularly the LRPPRC protein. LRPPRC is a key regulator of mitochondrial energy metabolism, promoting OXPHOS and ATP generation. However, when overexpressed, the LRPPRC acts as a tumor oncogene. Our analysis of SDHA and LRPPRC gene and protein expression patterns in precursor lesions and established ovarian cancer demonstrated that the upregulation of SDHA is accompanied by LRPPRC overexpression, notably in advanced tumors. Our novel findings highlight for the first time a potential functional interaction between SDHA and LRPPRC in the development and progression of ovarian malignancy. Importantly, our in vivo data showed that pharmacological inhibition of LRPPRC results in a lasting therapeutic benefit and can be an effective therapy in SDHA- and LRPPRC-overexpressing ovarian tumors. **Conclusions:** Overall, our study underlines an understudied role of concomitant overexpression of SDHA and LRPPRC in ovarian cancer pathogenesis, highlighting new paths for therapeutic development.

## Simple Summary

Cancer-specific metabolism contributes to tumor development and progression but also reveals metabolic vulnerabilities, offering an opportunity for therapeutic intervention. We examined the unique metabolism of ovarian cancer overexpressing succinate dehydrogenase subunit A (SDHA) and identified a distinctive vulnerability of these tumors to agents targeting key regulators of the OXPHOS pathway, particularly the LRPPRC protein. LRPPRC plays a central role in mitochondrial homeostasis and energy metabolism, promoting OXPHOS and ATP generation. Our analysis of transcriptomics data showed that the tumor-specific upregulation of SDHA is accompanied by LRPPRC overexpression, particularly in advanced ovarian cancer. Our in vivo data demonstrated that pharmacological blocking of LRPPRC’s function results in a long-term therapeutic benefit and can be an effective therapy in SDHA- and LRPPRC-overexpressing ovarian tumors. Overall, we strive to advance scientific knowledge by highlighting the understudied role of SDHA and LRPPRC in ovarian cancer and their potential utility as biomarkers of high-OXPHOS tumors to guide personalized cancer therapy.

## 1. Introduction

High-grade serous ovarian cancer (HGSOC) is an aggressive gynecological cancer with an extremely poor prognosis, largely owing to the scarcity of effective treatment [1,2]. Although HGSOC initially responds to cytoreductive surgery and standard chemotherapy, it remains incurable for most patients due to frequent recurrence and the development of chemotherapy resistance, leading to a 5-year survival rate below 40% [3,4]. New therapies are urgently needed, and biomarker-guided personalized treatment strategies hold great promise to improve ovarian cancer disease outcomes.

Metabolic reprogramming is a defining feature of cancer, allowing tumor cells to grow and proliferate at a faster rate and to survive cellular stress or anti-cancer treatment [5,6,7,8]. Over the past decade, significant progress has been made in understanding the diverse metabolic profiles of malignant tumors. Contrary to the long-held belief that cancer cells primarily rely on glycolysis (the Warburg effect) for energy, many tumor types have been found to preferentially utilize mitochondrial oxidative phosphorylation (OXPHOS) as a major energy source [9,10,11,12]. Cancers can broadly be categorized into two distinct metabolic subgroups: high-OXPHOS and low-OXPHOS tumors. The high-OXPHOS tumors show robust mitochondrial respiration, relying on the TCA cycle flux for their energy demands and biosynthetic needs, while low-OXPHOS tumors exhibit an enhanced glycolytic metabolism [9,10]. Studies show that mitochondrial energy metabolism plays a significant role in driving the aggressiveness and chemoresistance of ovarian cancer [9,11]. Moreover, metastatic ovarian tumors or cancers with stem cell-like features are highly metabolically active with increased mitochondrial respiration [6,11,12]. Thus, targeting key regulators of high-OXPHOS metabolism could be a promising therapeutic approach for ovarian cancer.

We discovered that a common feature of high-OXPHOS tumors is significantly overexpressed mitochondrial enzyme succinate dehydrogenase subunit A (SDHA), which is particularly prevalent in ovarian carcinoma (~20% of cases) [13]. Succinate dehydrogenase plays a critical role in cellular energy metabolism through its dual role in the TCA cycle and as a part of the electron transport chain (ETC). Specifically, SDHA acts within mitochondrial complex II, coupling the oxidation of succinate to fumarate with the reduction of ubiquinone to ubiquinol, directly connecting the TCA cycle with ETC [14]. In our previous work, mechanistic studies revealed that SDHA upregulation in ovarian cancer is associated with robust mitochondrial OXPHOS and ATP production, which are essential for the viability of tumor cells. In our earlier research, to identify compounds that selectively target ovarian cancer cells overexpressing SDHA, we screened a library of 64 anti-metabolic agents. Using this approach, shikonin emerged as the most effective agent in selectively eliminating SDHA-overexpressing ovarian tumor cells [13]. Shikonin is an herbal medicine that exhibits diverse therapeutic effects. Shikonin has been investigated as a potential anti-cancer agent and is recognized for its anti-metabolic activities, such as the inhibition of glycolysis and glutamine metabolism [15,16,17]. However, the underlying mechanism of critical sensitivity of SDHA-overexpressing tumors to shikonin is unknown due to shikonin’s numerous biological activities and the incompletely understood molecular targets [15,16]. The goal of this study is to address this gap in knowledge by investigating the metabolic vulnerabilities and mechanisms of high sensitivity of SDHA-overexpressing tumors to shikonin, which holds untapped opportunities for therapeutic intervention.

In this work, we showed that the aberrantly upregulated SDHA is a therapeutically relevant target in ovarian cancer. Our in vivo studies using mouse models of ovarian cancer showed that SDHA overexpression contributed to robust orthotopic tumor growth and metastasis, substantially reducing mouse survival. Further, using proteomics and metabolomics methods, we highlighted a central role of glutaminolysis, as well as increased activity of the TCA cycle coupled with mitochondrial OXPHOS in maintaining the high energy demands and biosynthetic needs of SDHA-overexpressing tumors. Next, we performed a drug target screening study to gain insight into the mechanism of the high sensitivity of SDHA-overexpressing tumors to shikonin. With this approach, we discovered that shikonin binds to and inhibits LRPPRC (leucine-rich pentatricopeptide repeat-containing) protein. LRPPRC is a multifunctional protein that plays an important role in cellular energy metabolism by inducing the synthesis of ETC complexes and promoting OXPHOS and ATP generation [18,19]. We next evaluated SDHA and LRPPRC gene and protein expression patterns in precursor lesions and advanced HGSOC samples and demonstrated that the tumor-specific upregulation of SDHA consistently coincides with LRPPRC overexpression, especially in advanced ovarian cancer. This work brings to light, for the first time, a possible functional relationship between SDHA and LRPPRC in the development and progression of ovarian cancer disease. Lastly, we demonstrated that shikonin treatment in vitro leads to a loss of OXPHOS activity, bioenergetic dysfunction, and death of SDHA- and LRPPRC-overexpressing ovarian cancer cell lines. In vivo, shikonin showed the most potent antitumor efficacy in high-SDHA, high-LRPPRC PDX models and limited efficacy in PDX models with low SDHA and LRPPRC expression.

Altogether, our study demonstrated that tumors overexpressing SDHA and LRPPRC are uniquely vulnerable to agents disrupting key regulators of the OXPHOS pathway, with LRPPRC being a critical target. Importantly, our findings revealed that the concomitant overexpression of SDHA and LRPPRC could be considered as novel biomarkers for identifying tumors that rely on high OXPHOS metabolism, offering a potential avenue for tailoring personalized cancer treatments.

## 2. Materials and Methods

### 2.1. Source of Ovarian Cancer Cell Lines and PDX Tumor Models

All human ovarian cancer cell lines used in this work are commercial, including OVCAR3 (#HTB-161, ATCC), OVCAR4 (#OVCAR-4, NCI-DTP), OVCAR8 (#OVCAR-8, NCI-DTP), and OVSAHO (#JCRB1046, JCRB). Normal human fallopian tube cell lines (FT190 and FT194) were developed by Dr. Drapkin from human fallopian epithelial cells and gifted to us [20]. Mouse ovarian cancer cell lines such as BPPNM, PPNM, SPCA, BPCA, and KPCA were derived from murine fallopian tube epithelium (mFTE) by Dr. Weinberg group and gifted to us [21]. Normal human fallopian tube tissues and established PDX tumor models were obtained from the PDX-PCT core facility at OMRF [22]. Additional information regarding cell line maintenance and validation is provided in Supplementary Figure S1A.

### 2.2. Soft Agar Colony Formation Assay

Soft agar colony formation assay was performed as previously published [13]. Briefly, cells were cultured in 6-well plates (4 × 10^4^ cells per well) in a mixture of 0.6% DifcoTM Noble Agar (#214220, BD Biosciences, Franklin Lakes, NJ, USA) and their respective media, which were added on top of a layer of 1% noble agar in culture medium. Cells were cultured for 10 days and then stained overnight with 200 µL of nitroblue tetrazolium chloride (#VWR0329, VWR Chemicals, Radnor, PA, USA). The visible colonies were photographed using a Leica 205 FCA microscope (Leica Microsystems Inc., Deerfield, IL, USA). Colony number was estimated using Image J, version 1.52a.

### 2.3. Generation of Lentiviruses and Cell Transduction

To generate ovarian cancer cell lines conditionally overexpressing SDHA, we used cell lines with low SDHA expression and conditionally overexpressed the SDHA coding sequence by transducing the cells with pLentiTRE/rtTA-SDHA lentivirus, as described previously [13]. The SDHA expression was induced by the addition of 100 ng/mL of doxycycline (dox) for 24 h. To knock down the SDHA gene, we used validated shRNA clones from Sigma-Aldrich (St. Louis, MO, USA) including TRCN0000028085 clone for human cell lines and TRCN0000346208 clone for mouse cell lines. To knock down the LRPPRC gene, we used TRCN0000295840 clone for human cell lines and TRCN0000216868 clone for mouse cell lines. The respective shRNAs were cloned into pLKO.1-puro lentiviral vector (Sigma-Aldrich, St. Louis, MO, USA). Recombinant lentiviruses were produced in HEK293T cells according to standard protocols [23]. Ovarian cancer cell lines were then infected with lentiviruses containing the shRNA against SDHA or LRPPRC or control shRNA with a scrambled sequence, followed by selection with puromycin, as described previously [23].

### 2.4. 3T5 Cell Proliferation Assay

The 3T5 cell proliferation assay was performed by plating 5 × 10^5^ cells per 10 cm tissue culture plate (each cell line was set up in triplicate), followed by counting and re-plating at the same density every 3 days for 13 days. Population doubling time was calculated using the formula ln(post-3-day cell count/5 × 10^5^)/ln(2). The given population doubling time was added to the cumulative doubling time of the previous count.

### 2.5. WES (ProteinSimple)

Cells or tumors were homogenized and lysed in Buffer B (25 mM Tris-HCl, pH 7.5, 0.42 M NaCl, 1.5 mM MgCl_2_, 0.5 mM EDTA, 1 mM DTT, 25% sucrose, 1 mM Na_3_VO_4_, and 1× protease inhibitor cocktail, #786-331, G-Biosciences, St. Louis, MO, USA) on ice for 15 min, followed by centrifugal clearing at 4 °C for 10 min at 10,000 rpm to recover whole-cell lysates. For analysis of proteins using a capillary electrophoresis-based protein analysis system (WES; ProteinSimple, San Jose, CA, USA), cellular proteins (0.5 mg/mL) were separated and visualized using the standard instrument protocol. Primary antibodies used were GAPDH (1:300, #sc-25778) from Santa Cruz Biotechnology (Santa Cruz, CA, USA); α-Tubulin (1:25, #2144), SDHA (1:50, #11998), cleaved PARP (1:50, #9541), and COX1/MT-CO1 (1:50, #55159) from Cell Signaling Technology (Danvers, MA, USA); and LRPPRC (1:50, # ab259927) from Abcam Inc. (Waltham, MA, USA). Anti-rabbit secondary antibodies were included in the Wes-Rabbit (12-230 kDa) Master Kit (#PS-MK14, ProteinSimple, San Jose, CA, USA).

### 2.6. Quantification of Protein Abundance Using High-Resolution Accurate Mass Spectrometry (HRA-MS)

We used quantitative HRA-MS method to measure and compare protein abundance in several key metabolic pathways between normal human fallopian tube samples and ovarian PDX with and without SDHA overexpression. Detailed information about the HRA-MS methodology is provided in Supplementary Figure S1A.

### 2.7. Metabolic Flux of Stable Isotope Labelled [U^13^C]-Glucose and [U^13^C]-Glutamine

Stable-isotope tracing studies were performed in the Metabolic Phenotypic Core facility at OMRF. Detailed protocol is provided in Supplementary Figure S1B.

### 2.8. Extraction of Shikonin-Bound Cellular Proteins

Shikonin or vehicle control was conjugated with epoxy-activated Sepharose 6B (ES6B), #95016-858, from Cytiva, Marlborough, MA, USA, which is a pre-activated medium for immobilization of various ligands. The shikonin-bound or vehicle-bound proteins were provided to the Multiplexing Protein Analysis Core Facility of the Oklahoma Nathan Shock Center to perform mass spectrometry-based drug discovery study. Detailed protocol is provided in Supplementary Figure S1B.

### 2.9. Mass-Spectrometry-Based Drug Discovery Study to Identify Shikonin’s Molecular Targets

Identification of shikonin’s molecular targets was performed in the Protein Analysis Laboratory at OMRF. Precipitates containing shikonin-bound proteins or proteins present in vehicle control samples were mixed with SDS–PAGE sample buffer and boiled for 10 min. The samples were loaded and separated by 1.5cm on a 10% SDS–polyacrylamide gel. The gel was stained with GelCode blue. The whole lane was digested in gel and analyzed via data-dependent analysis using the QEx Plus tandem mass spectrometry system (Thermo Thermo Fisher Scientific, Waltham, MA, USA). Proteins were identified by searching the human RefSeq database using Mascot (version 2.5.1).

### 2.10. Modified Pull Down Assay Coupled with WES(ProteinSimple) to Validate Shikonin Binding to LRPPRC

To determine if shikonin can directly bind to LRPPRC protein, shikonin-conjugated epoxy-activated Sepharose-6B (ES6B) or control ES6B-DMSO were incubated with human recombinant c-Myc/DDK-tagged LRPPRC protein (#TP316747, ORiGene, Rockville, MD, USA). Specifically, 1 ng of recombinant LRPPRC was mixed with 50 µL of ES6B-shikonin or ES6B-DMSO (prepared as described above) and filled up to 400 µL with Binding Buffer. The mixtures were incubated for 16 h at 4 °C with constant and gentle stirring. After incubation, precipitates were spun for 2 min, 14,000 rpm at 4 °C, and supernatants were gently removed and discarded. Precipitate pellets were washed 4 times with 800 µL of chilled RIPA Buffer by spinning the tubes for 2 min at 14,000 rpm and discarding supernatant. Next, 80 µL of 2× SDS Buffer was added to precipitate pellets, and samples were boiled for 10 min. Samples were centrifuged (10 min at 10,000 rpm at 4 °C), and supernatants were collected. Lastly, supernatants were processed by WES(ProteinSimple) to visualize eluted shikonin-bound proteins (recombinant LRPPRC) according to a standard instrument protocol. Primary antibodies used were LRPPRC (1:50, #ab259927) from Abcam Inc. and DDK rabbit monoclonal antibody (1:50, #TA592569S) from ORiGene. Secondary antibodies were included in a Wes Master Kit (PS-MK14, ProteinSimple).

### 2.11. Animal Experiments

All animal procedures were approved by the OMRF’s Institutional Animal Care and Use Committee. To determine the effect of SDHA upregulation on orthotopic tumor growth in immunocompetent mice, 6-week-old female C57BL/6J mice (#000664, Jackson Laboratory, Bar Harbor, ME, USA) were implanted with BPPNM, BPPNM-SDHA-KD, or SPCA tumor cells. Specifically, 5 × 10^5^ of respective tumor cells were suspended in 50% matrigel/50% Hanks’ Balanced Salt Solution (HBBS) and injected into ovarian bursa. Matrigel Corning^TM^ (#CLS354234) was obtained from MilliporeSigma, Burlington, MA, USA; HBBS (#14175095) was purchased from Thermo Fisher Scientific, Waltham, MA, USA). Mice were monitored for tumor growth, metastases, and ascites development via palpation and body weight measurement. Moribund mice were euthanized, and overall survival was analyzed using Kaplan–Meier curves and the two-sided log-rank test.

In different experiment, to evaluate therapeutic response to shikonin in vivo, C57BL/6J female mice were implanted with SDHA-overexpressing BPPNM model as described above. When tumors became palpable (~100 mm^3^ volume), animals were randomized and treated with vehicle control, shikonin (10 mg/kg, 3×/week), or carboplatin (50 mg/kg, 1×/week) + paclitaxel (10 mg/kg 1×/week). Mice were monitored for tumor growth, response to treatment, and overall survival, which was evaluated and compared between the groups using Kaplan–Meier method. To determine shikonin’s efficacy in vivo using human ovarian PDX models, 6-week-old female NRG mice (#007799, Jackson Laboratory) were used. PDXs were implanted subcutaneously (SQ) into the dorsal flank using our routine procedures [22]. Mice with established tumors of 100 mm^3^ volume were randomized and started 4-week treatment with vehicle control, shikonin (10 mg/kg, 3×/week), or cisplatin (5 mg/kg, 1×/week) + paclitaxel (10 mg/kg 1×/week), followed by an 8-week follow-up period to assess tumor growth and response to treatment or eventual disease relapse. Tumor volumes were calculated using the formula ½ (Length × Width^2^). At the endpoint, animals were humanely euthanized via CO_2_ inhalation.

### 2.12. Dose Response Assays

Exponentially growing cells were treated in vitro with indicated drugs, followed by an MTT assay to measure cell viability using the Quick Cell Proliferation Assay kit II (#K302-500, BioVision, Milpitas, CA, USA). Briefly, cells were seeded in a 96-well plate at a density of 5000 cells per well. After 24 h of culture, the cells were exposed to desired concentration of drug(s) or vehicle control for 4 days. Next, cells were incubated with the WST reagent for 2 h, and absorbance was determined at 450 nm. Absorbance measurements were normalized to the drug vehicle control wells.

### 2.13. Drugs and Reagents

Shikonin (#S8279) was purchased from Selleck Chemicals, Houston, TX, USA. For in vivo studies, desired concentration of shikonin was prepared by adding solvents to the shikonin powder individually, 2% DMSO, 40% PEG300, 5% Tween80, and ddH_2_O. Shikonin solution was mixed until clear, protected from light, and administered within 30 min after preparation. Cisplatin (#NDC67457-0424, Alvogen, Morristown, NJ, USA), carboplatin (#NDC16729-0295, Intas Pharmaceuticals Limited, Ahmedabad, India), and paclitaxel (Actavis Generics, Parsippany-Troy Hills, NJ, USA) were purchased from University of Oklahoma Pharmacy and diluted to desired concentrations in saline solution or PBS, respectively.

### 2.14. In Silico Analysis of SDHA and LRPPRC Gene Expression in Human HGSOC Samples

An analysis of SDHA and LRPPRC gene expression profiles in human tissue specimens representing consecutive stages of HGSOC development was performed with the use of GeoMx spatial transcriptomics data. The GeoMx dataset is publicly available via cBioPortal (www.cbioportal.org; last accessed on 3 April 2025) in the “Ovarian Cancer (Gray Foundation, Cancer Discov 2024)” category. All sample processing and sequencing were performed by the Dana Farber Sequencing facility. The quality control and the Quartile-3 normalization of the initial data set were performed as suggested by NanoString using GeoMx DSP software (v2.1), NanoString (version 3.1.0.221). Detailed information about patients’ cohorts, sample collections, tissue processing, and spatial transcriptomic profiling using the Nanostring GeoMx platform is found elsewhere [24].

### 2.15. Measurement of OCR and ATP Production Rate by Seahorse

The Seahorse XFe24 Extracellular Flux Analyzer (Agilent Technologies, Santa Clara, CA, USA) was used to assess bioenergetic profiles of ovarian cancer cell lines. The Seahorse XF Cell Mito Stress assay (#103015-100) was used to quantify mitochondrial respiration (oxygen consumption rate, OCR) and ATP production rate. Detailed protocol is included in Supplementary Figure S1C.

### 2.16. Statistical Analysis

In vitro experiments were performed three times and in triplicate when applicable. Values are presented as mean ± SD or as mean ± SEM. Statistical analysis of in vitro assays or in vivo data was performed using unpaired t-test, multiple t-test, or analysis of variance (ANOVA), followed by Tukey’s multiple comparisons test whenever applicable. *p* < 0.05 was considered significant. For mouse survival, Kaplan–Meier survival curves were made with P value generated from log-rank test. Statistical analysis was performed using GraphPad Prism 10.4.0 (621) Software (San Diego, CA, USA).

## 3. Results

### 3.1. SDHA Overexpression Promotes Proliferation and Survival of Ovarian Tumor Cells in Suspension Cultures

**Figure 1:**
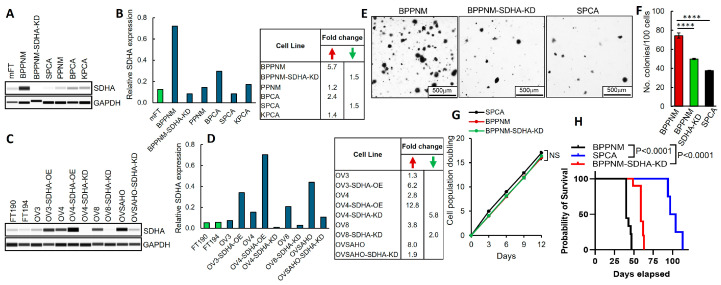
The effect of SDHA overexpression on ovarian cancer cell proliferation and survival evaluated in human and mouse tumor models. (**A**) WES analysis of mouse normal fallopian tubes “mFT” and ovarian cancer cell lines assayed for SDHA and loading control GAPDH. (**B**) Graph represents normalized quantification of SDHA protein expression in cell lines shown in (**A**) using Compass for SimpleWestern Software (version 4.1.0.). Table shows fold change of SDHA expression in mouse ovarian cancer cell lines relative to the expression of SDHA in normal mFTs. (**C**) WES analysis of human fallopian tube cell lines (FT190, FT194) and human ovarian cancer cell lines with and without overexpression (OE) or knockdown (KD) of SDHA assayed for SDHA and GAPDH. (**D**) Fold change of SDHA expression in human ovarian cancer cell lines shown in (**C**) as relative to the expression of SDHA in normal human FTs. (**E**) Images represent anchorage-independent growth and colony formation of mouse ovarian cancer cell lines overexpressing SDHA (BPPNM), those depleted of SDHA via shRNA-mediated knockdown (BPPNM-SDHA-KD), and those with naturally low SDHA levels (SPCA). The SDHA-OE significantly improves colony formation and survival of ovarian cancer cells in suspension cultures. (**F**) Number of cell colonies shown in (**E**) was quantified (one-way ANOVA; **** = *p* < 0.0001). (**G**) Cell proliferation in vitro was assessed via 3T5 cell doubling assay in cell lines with and without SDHA overexpression (one-way ANOVA). The SDHA expression has no effect on cell proliferation in adherent cell cultures. (**H**) Kaplan–Meier curves showing % probability of survival of mice injected into ovary with BPPNM, BPPNM-SDHA-KD, or SPCA cells. Mice implanted with SDHA-OE tumors (BPPNM) showed significantly reduced overall survival due to rapid tumor progression. (**A**,**C**) Uncropped blots are shown in Supplementary Figure S1.

Thus far, we have studied the effects of SDHA overexpression solely with the use of human ovarian cancer models [13]. Such tumor models require immunocompromised mouse strains to be grown in vivo, lacking the functional immune system, which is an essential component of the tumor microenvironment (TME). Here, to expand our studies exploring the impact of SDHA overexpression on tumor biology, we obtained innovative ovarian cancer cell lines derived from murine fallopian tube epithelium (mFTE) from Dr. Weinberg [21]. These mFTE cell lines harbor patient-relevant mutant genotypes and form tumors in immunocompetent C57BL/6J mice to fully represent the immunocompetent TME in vivo (Supplementary Figure S1D–F). First, we quantified SDHA levels in mFTE cell lines and normal mouse fallopian tube (mFT) cells as controls using WES (capillary electrophoresis-based protein analysis system from ProteinSimple). We identified BPPNM cell line with a significant upregulation (5.7-fold increase) of SDHA expression when compared with mFT cells. Other mFTE cell lines showed low SDHA expression similar to that observed in normal fallopian tubes or BPPNM cells with SDHA knockdown (BPPNM-SDHA-KD), Figure 1A,B and Supplementary Figure S1G.

We also expanded our panel of genetically engineered human ovarian cancer cell lines with conditional (dox inducible) SDHA overexpression (SDHA-OE) or stable SDHA knockdown (KD), as well as established human fallopian tube cell lines (FT190 and FT194) as controls (Figure 1C,D and Supplementary Figure S1H). The SDHA expression was very low in normal human fallopian tubes (FT190 and FT194) but became significantly increased (2.8-8.0-fold increase) in ovarian cancer cell lines that naturally overexpress SDHA (OVCAR4, OVCAR8, and OVSAHO), as shown in Figure 1C,D. The conditional SDHA overexpression increased SDHA levels 6.2-fold in OVCAR3-SDHA-OE and 12.8-fold in OVCAR4-SDHA-OE when compared with human fallopian tubes, while SDHA-KD reduced SDHA expression 2.0 or 5.8-fold in OVCAR8 and OVCAR4 cells, respectively (Figure 1C,D). The SDHA expression in OVSAHO-SDHA-KD is similar to that observed in fallopian tubes.

Next, using mFTE cell lines, we determined the effect of SDHA overexpression on anchorage-independent growth and cell survival. We observed that the BPPNM cells endogenously overexpressing SDHA showed significantly higher numbers of tumor cell colonies than the SPCA cell line with naturally very low SDHA expression or the isogenic BPPNM cell line with SDHA knockdown (Figure 1E,F). However, when we measured the cumulative population doubling of cells in adherent cell cultures in vitro, we did not observe any changes in cell proliferation; the cells proliferated at a similar rate regardless of SDHA overexpression status (Figure 1G). We made similar observations in various human ovarian cancer cell lines with and without SDHA overexpression or knockdown, where the SDHA upregulation promotes cell proliferation and survival in suspension cultures but not in adherent cell cultures (Supplementary Figure S2).

Collectively, our findings show that the SDHA overexpression promotes cell survival and proliferation in suspension conditions in both human and mouse ovarian cancer models, which is a key feature of patients’ ovarian tumors that are able to survive, proliferate, and metastasize in suspension in peritoneal fluid.

### 3.2. SDHA Upregulation Promotes Orthotopic Tumor Growth in Immunocompetent Mouse Models of Ovarian Cancer

We evaluated the effect of SDHA overexpression on tumor growth, metastasis, and mouse survival in vivo using immunocompetent mouse models of ovarian cancer. We selected the SDHA-overexpressing BPPNM model and low-SDHA models (SPCA and BPPNM-SDHA-KD, Figure 1A). First, we carried out a pilot in vivo experiment using C57BL/6J female mice, where tumor cells were implanted into the mouse ovary to recapitulate early stages of ovarian cancer development or into the peritoneum to model advanced tumor progression. The intraovarian route of cancer cell implantation resulted in the development of large tumors that disseminated throughout the abdominal cavity, invading the omentum, intestines, and diaphragm. Intraperitoneal cancer cell inoculation led to the generation of a large number of smaller tumors invading the omentum, diaphragm, intestines, liver, and stomach, as well as robust ascites. Both routes of tumor implantation recapitulated typical HGSOC histopathology (Supplementary Figure S3A–E). We observed similar results regardless of route of tumor implantation (intraovarian or intraperitoneal), where BPPNM tumors developed significantly faster, generating larger tumor masses and robust ascites when compared with SPCA tumors, which substantially reduced mouse survival (Supplementary Figure S3A–E).

Next, we performed a larger in vivo study using a pair of isogenic cell lines with and without SDHA knockdown (BPPNM and BPPNM-SDHA-KD) and an unrelated SPCA cell line with naturally low SDHA levels. We implanted respective cancer cells into the C57BL/6J female mouse ovary and monitored the animals for tumor development. Similarly, as in the pilot study, the SDHA-overexpressing tumors (BPPNM) showed increased tumorigenic potential reflected as rapid tumor development and spreading when compared with low-SDHA tumors (SPCA) or BPPNM-SDHA-KD, which significantly reduced mouse survival (Figure 1H). A macroscopic evaluation of tumor burden at necropsy showed that tumor masses, metastatic lesions, and/or ascites were similar in BPPNM and BPPNM-SDHA-KD tumor-bearing mice. In contrast, mice inoculated with SPCA cancer cells developed smaller tumors and fewer metastatic lesions (Supplementary Figure S3F–H).

### 3.3. The Majority of Differentially Expressed Proteins Between Normal Human Fallopian Tubes and Ovarian PDXs, Particularly PDXs with SDHA Overexpression, Are Components of the TCA Cycle

**Figure 2:**
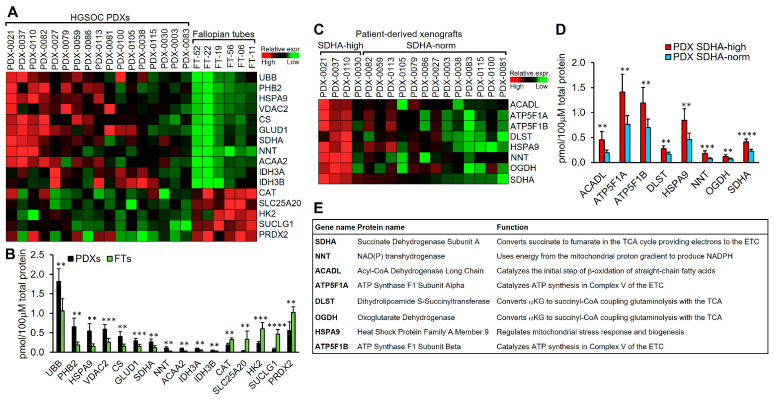
Analysis of protein content of metabolic pathways in ovarian PDXs and normal hFTs. (**A**) A heatmap representing differentially expressed metabolic proteins between ovarian PDXs and hFTs identified via HRA-MS analysis (unpaired *t* test). (**B**) Graph shows levels of differentially expressed proteins between PDXs and hFTs identified by HRA-MS (unpaired t test). A large proportion of upregulated proteins in PDXs are components of the TCA cycle. (**C**) A heatmap illustrating differentially expressed metabolic proteins between SDHA-overexpressing PDXs vs. PDX with normal SDHA levels detected by HRA-MS (unpaired *t* test). (**D**) Differentially expressed proteins between SDHA-overexpressing PDXs vs. PDX with normal SDHA levels (unpaired *t* test). The majority of overexpressed proteins are integral to the function of the TCA cycle and ETC. (**E**) Table shows full name and function of differentially expressed proteins listed in (**D**). (**B**,**D**) Asterisks indicate level of statistical significance: ** *p* ≤ 0.01, *** *p* ≤ 0.001, **** *p* ≤ 0.001. Detailed information about HRA-MS data is provided in Supplementary Figure S4.

We interrogated metabolic changes that occur as ovarian cancer develops from the fallopian tube to invasive HGSOC. We also defined differences in metabolic protein content between SDHA-overexpressing tumors and those with normal SDHA levels. We used our established HGSOC PDX models [22] and normal human fallopian tubes (hFTs) and performed an absolute quantification of metabolic proteins by HRA-MS [25]. We evaluated the protein content of major metabolic pathways, including β-oxidation of fatty acids, gluconeogenesis, glutamine metabolism, glycolysis, mitochondrial function and ETC, oxidative stress response, and the TCA cycle. We measured a total of 85 proteins (represented by unique gene symbols), as shown in Supplementary Figure S4A. First, we defined changes in protein content between ovarian PDXs and hFTs and identified 16 differentially expressed proteins, the majority of which were involved in the oxidative stress response pathway and the TCA cycle (Figure 2A,B and Supplementary Figure S4B,C). Among metabolic pathways, the TCA cycle protein content was the most significantly altered between hFTs and ovarian PDXs. Specifically, we identified a significantly increased protein expression of citrate synthase (CS), succinate dehydrogenase (SDHA), and isocitrate dehydrogenases (IDH3A and IDH3B) in ovarian PDXs vs. hFTs. These are key enzymes in the TCA cycle, which governs cellular energy production and generates metabolic intermediates for biosynthetic pathways [26]. Furthermore, in PDX tumors, we observed the upregulation of several proteins within the oxidative response pathway (UBB, PHB2, and HSPA9) and the mitochondria function pathway (NNT and VSAC2) that play an essential role in cellular antioxidant defense or function as chaperone proteins to stabilize mitochondrial respiratory enzymes and maintain mitochondrial integrity [27].

Lastly, we detected the overexpression of mitochondrial enzymes such as acetyl-CoA acyltransferase 2 (ACAA2) and glutamate dehydrogenase 1 (GLUD1) in PDXs vs. hFTs. ACAA2 catalyzes the final step of mitochondrial β-oxidation of fatty acids, which is a catabolic pathway where fatty acids are metabolized to produce energy [28]. GLUD1 is a key enzyme in the glutaminolysis pathway, converting glutamate to αketoglutarate (αKG), which is an important intermediate in the TCA cycle [29]. Among proteins showing increased abundance in normal hFTs compared to ovarian PDXs, we detected catalase (CAT), peroxiredoxin 2 (PRDX2), hexokinase 2 (HK2), solute carrier family 25 member 20 (SLC25A20), and succinate-CoA ligase GDP/ADP-forming subunit alpha (SUCLG1). CAT and PRDX2 are antioxidant enzymes commonly found in normal fallopian tubes and are essential for normal reproductive physiology [30]. HK2 is known to be upregulated in various cancers, promoting tumor progression; however, high activity of hexokinases has also been detected in endosalpinx (mucous membrane lining the fallopian tubes), where glucose is a major metabolic fuel [31]. SCL25A20 is a carrier protein transporting acylcarnitines into the mitochondrial matrix for the β-oxidation of fatty acids. SLC25A20 is frequently downregulated in cancer, which leads to a suppression of β-oxidation of fatty acids, promoting tumor growth and metastasis [32,33]. We also observed the downregulation of SUCLG1 in ovarian PDXs catalyzing the conversion of succinyl-CoA to succinate in the TCA cycle. It has been shown in certain cancers that SUCLG1 becomes downregulated in tumor tissue, even though the remaining TCA cycle components are upregulated, which indicates additional unexplored functions of SUCLG1 [34,35].

In the next step, we identified SDHA-overexpressing PDX models, which showed SDHA protein expression greater than the 75th percentile value of SDHA levels in remaining PDXs. Our HRA-MS results demonstrated that the SDHA-overexpressing PDXs are characterized by the significantly increased expression of proteins, primarily in the TCA cycle and mitochondrial ETC (Figure 2C–E and Supplementary Figure S4D,E). The high-SDHA PDXs showed significantly elevated levels of SDHA as expected, αketoglutarate dehydrogenase (αKGDH) components (DLST and OGDH), and mitochondrial ATP synthase subunits (ATP5F1A and ATP5F1B) (Figure 2C–E). The αKGDH complex is the primary site of control of metabolic flux through the TCA cycle [36,37]. The αKGDH collaborates with glutaminolysis at the intersectional point to govern αKG levels for energy production and resources for macromolecule synthesis in cancer cells [38]. Additionally, the high-SDHA PDXs showed significantly upregulated NNT and HSPA9 proteins when compared with low-SDHA PDXs (Figure 2C–E), which are also among differentially expressed proteins between a whole panel of PDXs and normal hFTs (Figure 2A,B). Lastly, high-SDHA PDXs show significantly increased levels of Acyl-CoA Dehydrogenase Long Chain (ACADL), an enzyme that catalyzes the initial step of β-oxidation of fatty acids, which plays an essential role in mitochondrial energy production (Figure 2C–E).

Altogether, our proteomics data highlight the significance of the TCA cycle metabolic pathway coupled with ETC in supporting the metabolic requirements of SDHA-overexpressing ovarian tumors.

### 3.4. SDHA-Overexpressing Ovarian Cancer Cells Rely on Glutaminolysis to Maintain an Increased TCA Cycle Flux and OXPHOS Activity

To gain insight into the metabolic flux of glucose (Glu) and glutamine (Gln) in SDHA-overexpressing ovarian cancer cells and to specifically determine if glutamine replenishes TCA cycle intermediates, we performed stable-isotope tracing. We used the uniformly labeled stable isotopes [U^13^C]glucose and [U1^3^C]glutamine to determine their contribution to metabolic intermediates within glycolysis and the TCA cycle pathway via quantification of isotopic enrichment using liquid chromatography-quadrupole/time-of-flight (LC-Q/TOF) mass spectrometry. The analysis of glycolysis flux in OVCAR4-SDHA vs. OVCAR4 control cells revealed that glycolytic intermediates were rapidly labeled with [U^13^C]glucose. At 30 min of labeling, the m+3 enrichment of glycolytic intermediates reached saturation. Furthermore, the m+3 isotopomer enrichment was very similar in both cell lines (Supplementary Figure S5A,B). These data indicate that SDHA overexpression does not affect glycolytic flux. Next, we quantified the relative metabolite abundance, which included both ^13^C-labeled and ^12^C-unlabeled intermediates, within the glycolytic pathway. We observed an increased abundance of glycolytic intermediates downstream of glucose-6 phosphate (G6P), including F6P, F1, 6BP, DHAP, PEP, and 3-PGA in OVCAR4-SDHA cells when compared with controls (Supplementary Figure S5C–E). Since both cell lines showed similar glycolytic flux, the increased pool of glycolytic intermediates in OVCAR4-SDHA cells could be associated with decreased flux of G6P into other pathways, such as the pentose phosphate pathway (PPP).

**Figure 3:**
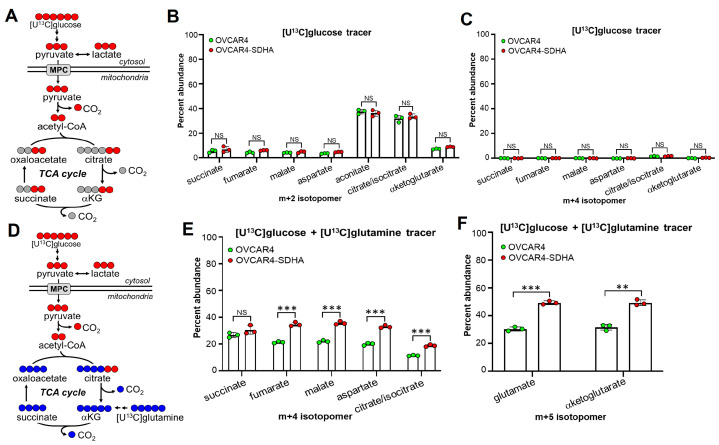
Analysis of TCA cycle fluxes of [U^13^C]glucose and [U^13^C]glutamine. (**A**) The diagram represents the flux of metabolites from a single tracer [U^13^C]glucose via the TCA cycle in OVCAR4 cells. Red circles indicate ^13^C, gray circles indicate ^12^C. (**B**) The percentage abundance of the m+2 isotopomers within the TCA cycle metabolite pool in OVCAR4-SDHA vs. OVCAR4 control cells following tracing of [U^13^C]glucose (unpaired *t* test). The contribution of Glu-derived carbon to the TCA cycle is primarily reflected in the production of citrate, isocitrate, and aconitate at comparable levels in both OVCAR4-SDHA and OVCAR4 cells. (**C**) The percentage abundance of the m+4 isotopomers within the TCA cycle in OVCAR4-SDHA vs. OVCAR4 control cells following tracing of [U^13^C]glucose (unpaired *t* test). Minimal m+4 labeling of TCA cycle intermediates with the [U^13^C]glucose suggests that glucose contributes only marginally to the production of these metabolites. (**D**) Flux of metabolic intermediates from both tracers [U^13^C]glucose and [U^13^C]glutamine via TCA cycle in OVCAR4 cells. Red circles indicate ^13^C derived from glucose; blue circles indicate ^13^C derived from glutamine. (**E**) The percentage abundance of the m+4 isotopomers within the TCA cycle in OVCAR4-SDHA vs. OVCAR4 controls following parallel tracing of [U^13^C]glucose and [U^13^C]glutamine (unpaired *t* test). The increased m+4 labeling with both tracers [U^13^C]glucose and [U^13^C]glutamine vs. [U^13^C]glucose alone, shown in "C", indicates that m+4-labeled metabolites are derived primarily from glutamine, particularly in OVCAR4-SDHA cells. (**F**) The percentage abundance of the m+5 isotopomers within the TCA cycle in OVCAR4-SDHA vs. OVCAR4 cells following parallel tracing of [U^13^C]glucose and [U^13^C]glutamine (unpaired *t* test). The increased m+5 labeling with both tracers vs. m+2 or m+4 labeling with [U^13^C]glucose alone (shown in (**B**) and (**C**), respectively) indicates a markedly enhanced Gln flux into the TCA cycle, which is significantly increased in SDHA-overexpressing tumor cells vs. controls. (**B**,**C**,**E**,**F**) Levels of statistical significance: ** *p* ≤ 0.01, ***p ≤ 0.001, NS not significant. Additional information is provided in Supplementary Figure S5.

As a next step, we analyzed the flux of TCA cycle intermediates with either a single tracer [U^13^C]glucose or double tracers ([U^13^C]glucose and [U^13^C]glutamine (Figure 3 and Supplementary Figure S5F,G). Analysis of the TCA cycle metabolite pool with [U^13^C]glucose revealed similar enrichment of m+2 labeled TCA cycle intermediates in OVCAR4-SDHA and OVCAR4 cells. The m+2 labeling of citrate, isocitrate, and aconitate was higher than the m+2 labeling of succinate, fumarate, malate, and aspartate in both cell lines (Figure 3B). These data indicate that glucose carbon contribution to the TCA cycle is largely associated with the generation of citrate, isocitrate, and aconitate, which are key metabolites linking glycolysis and lipid metabolism and promoting de novo lipid biosynthesis. Further, we observed a minimal labeling (0–2%) of m+4 TCA cycle intermediates with a [U^13^C]glucose (Figure 3C). This indicates that glucose is used as a source of acetyl-CoA for entry into the TCA cycle, but the subsequent turns of the cycle are either diluted by unlabeled carbon sources entering the cycle, or labeled intermediates are being rapidly siphoned off for other metabolic pathways, preventing the accumulation of multiply-labeled oxaloacetate (OAA) for subsequent m+4 production. This is in contrast to the [U^13^C]glucose and [U^13^C]glutamine double labeling experiment, where the m+4 labeling ranged from 12 to 36% (Figure 3E). In addition, we noted significantly increased enrichment of m+4-labeled fumarate, malate, aspartate, citrate, and isocitrate, as well as m+5-labeled α-ketoglutarate (αKG) and glutamate, in OVCAR4-SDHA cells compared to OVCAR4 cells (Figure 3E,F).

Together, these data indicate that the majority of m+4- and m+5-labeled TCA cycle intermediates with both tracers ([U^13^C]glucose and [U^13^C]glutamine) were produced by [U^13^C]glutamine, and that glutamine showed significantly increased flux into the TCA cycle in tumor cells with SDHA overexpression. Quantifying total intermediates (which included both ^13^C- and ^12^C-intermediates), we also noted a higher abundance of citrate, isocitrate, and aconitate in SDHA-overexpressing cells vs. controls in experiments (Supplementary Figure S5F,G). Since SDHA-overexpressing cells show increased glutamine-derived m+4 and m+5 carbon flux (Figure 3E,F) and similar glucose-derived m+2 carbon flux into the TCA cycle when compared with control cells (Figure 3B), this indicates that the increased pool of citrate, isocitrate, and aconitate metabolites in high-SDHA cells could be associated with decreased flux of those intermediates into the de novo lipid biosynthesis pathway. Consequently, limited citrate export for lipid production, combined with increased glutamine influx via anaplerotic reactions, likely contributes to the accumulation of citrate, isocitrate, and aconitate in SDHA-overexpressing tumor cells. These data agree with Seahorse OCR assays performed with a combination of glucose and glutamine, where SDHA overexpression significantly increases mitochondrial respiration and ATP production in OVCAR4-SDHA cells when compared with control OVCAR4 cells (Supplementary Figure S5I,J).

### 3.5. Identification of LRPPRC Protein as a Top Molecular Target of Shikonin

**Figure 4:**
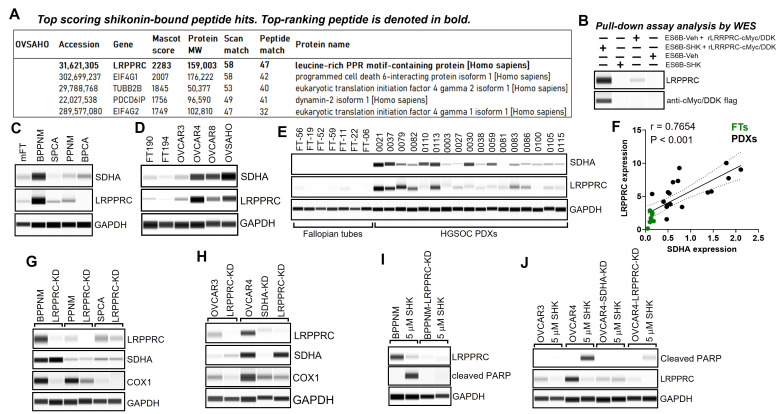
LRPPRC as a key molecular target of shikonin. (**A**) Representative results with the use of SDHA-overexpressing OVSAHO ovarian cancer cell line showing MASCOT score analysis of top-ranking shikonin-bound peptides (additional information is provided in Supplementary Figure S6. (**B**) Validation of a direct binding of shikonin to LRPPRC protein assessed via pull-down assay using shikonin-conjugated ES6B and OVCAR4 cell extracts with and without addition of recombinant LRPPRC protein tagged with c-terminal Myc-DDK tags. The assay validated that the shikonin binds with high affinity to LRPPRC. (**C**) WES analysis of SDHA and LRPPRC levels in mFTE cell lines vs. normal mouse fallopian tubes (mFT) shows concomitant expression of SDHA and LRPPRC (GAPDH, loading control). (**D**) WES showed marginal levels of SDHA and LRPPRC in human fallopian tubes (FT-190 and FT-194) and a high concomitant expression of SDHA and LRPPRC in human ovarian cancer cell lines. (**E**) WES detected marginal levels of SDHA and LRPPRC in hFTs and a high concomitant expression of SDHA and LRPPRC in a subgroup of HGSOC PDXs. (**F**) Assessment of Pearson correlation coefficient (r) revealed a strong positive correlation (r = 0.7654, *p* < 0.001) between SDHA and LRPPRC protein expression in HGSOC PDXs and hFTs. (**G**) The effects of LRPPRC-KD on SDHA and COX1 protein levels in mFTE cell lines assessed by WES. LRPPRC-KD leads to a loss of LRPPRC and COX1 expression but has no effect on SDHA protein levels. (**H**) The effects of LRPPRC-KD or SDHA-KD in human ovarian cancer cell lines. SDHA-KD is associated with loss of protein expression of both SDHA and LRPPRC, while LRPPRC-KD leads to a loss of LRPPRC and COX1 expression but has no effect on SDHA protein levels. (**I**) WES showing the expression of cleaved PARP (apoptosis marker), LRPPRC, and GAPDH (loading control) in mFTE cell lines treated with 5 μM dose of shikonin for 24 h in vitro. Shikonin treatment leads to a loss of LRPPRC expression and triggers apoptosis. (**J**) WES showing the expression of cleaved PARP, LRPPRC, and GAPDH in human ovarian cancer cell lines treated with 5 μM dose of shikonin for 24 h in vitro. Shikonin treatment leads to a loss of LRPPRC expression and triggers apoptosis. Uncropped blots are shown in Supplementary Figure S7.

We performed a mass spectrometry-based drug target discovery study to identify shikonin’s unexplored targets to determine the specific mechanism by which shikonin effectively eradicates SDHA-overexpressing tumor cells. We incubated ovarian cancer cell lysates with shikonin-conjugated Sepharose-6B to isolate shikonin-binding proteins, which were then analyzed using mass spectrometry (Supplementary Figure S6). We discovered that LRPPRC was a major shikonin-binding protein in SDHA-overexpressing cells, ranking among the top peptide hits in the MASCOT score analysis (Figure 4A). Further, we performed a pull-down assay coupled with WES (ProteinSimple) using shikonin-conjugated Sepharose-6B and cell extracts with and without the addition of cMyc/DDK-tagged recombinant LRPPRC protein. Shikonin-bound proteins were precipitated and analyzed using WES, validating our novel findings that shikonin binds with high affinity to the LRPPRC protein (Figure 4B and Supplementary Figure S7A). The LRPPRC protein emerged as a tumor oncogene with a central role in mitochondrial homeostasis and energy metabolism [18,19]. Since shikonin effectively eliminates SDHA-overexpressing tumor cells [13] while targeting LRPPRC, this suggests a potential unexplored interaction or dependence between SDHA and LRPPRC proteins.

### 3.6. SDHA and LPPPRC Transcript Levels Progressively Increase from Precancerous Lesions to Invasive HGSOC and Exhibit Concomitant Gene and Protein Expression Patterns

We analyzed GeoMx spatial transcriptomics data (available via cBioPortal) of human tissues collected at different stages of HGSOC development, including normal fallopian tube (FT), precursor lesions such as p53 signature and serous tubal intraepithelial carcinomas (STIC), and invasive ovarian carcinoma. We aimed to determine changes in SDHA and LRPPRC gene expression ranging from early precursor lesions to advanced-stage HGSOC to provide insight into the potential role of those molecules in the development and progression of ovarian cancer disease. Our findings revealed a shift toward significantly higher gene expression of both SDHA and LRPPRC when precursor lesions progressed to advanced ovarian cancer. Specifically, SDHA and LRPPRC expression was significantly lower in normal FT when compared with ovarian cancer (Supplementary Figure S8A). Furthermore, while there was no correlation between SDHA and LRPPRC expression in premalignant lesions (FT and p53 signature), in STIC lesions and invasive cancer, however, we observed an increasing positive correlation between the expression of those genes, which was particularly strong in invasive cancer (STIC, r = 0.2675, *p* = 0.0064; invasive cancer, r = 0.5671, *p* < 0.0001, Supplementary Figure S8B).

Next, we measured SDHA and LRPPRC protein expression in mouse and human ovarian cancer cell lines, HGSOC PDXs, and normal hFTs using WES(ProteinSimple) and observed a concomitant SDHA and LRPPRC protein expression in all examined tissues. The expression of both LRPPRC and SDHA was very low in normal fallopian tubes when compared with cancer samples (Figure 4C–E). Next, to determine the statistical significance of a correlation between SDHA and LRPPRC protein expression in HGSOC PDXs and hFTs, we calculated Pearson correlation coefficient (r) values. The results showed a strong positive correlation (r = 0.7654, *p* < 0.001) between SDHA and LRPPRC protein expression (Figure 4F). Next, to determine if SDHA or LRPPRC depletion affects each other’s protein expression, we knocked down (KD) either SDHA or LRPPRC in several mouse and human ovarian cancer cell lines. The results showed that the SDHA-KD is associated with a loss of protein expression of both SDHA and LRPPRC (Figure 4G,H), while LRPPRC-KD leads to a loss of LRPPRC expression (as expected) but has no effect on SDHA protein expression (Figure 4H). We also analyzed changes in the expression of COX1 (a subunit of Complex 4 of ETC) following SDHA-KD and LRPPRC-KD as a control, since COX1 expression is positively regulated by LRPPRC [18,39]. COX1 expression was suppressed in LRPPRC-KD cell lines or those with naturally low LRPPRC levels, as previously reported (Figure 4G,H) [39].

Collectively, these findings show that SDHA and LRPPRC expression progressively increases from precancerous lesions to invasive cancer, highlighting their potential as biomarkers and therapeutic targets for advanced HGSOC disease.

### 3.7. Shikonin Inhibits LRPPRC, Suppressing Mitochondrial Respiration, Which Leads to Bioenergetic Dysfunction and Death of Cancer Cells Overexpressing SDHA and LRPPRC

We investigated the effect of shikonin treatment in vitro on cellular metabolism and the survival of SDHA-overexpressing tumors in the presence and absence of LRPPRC expression (genetic knockdown). Our data revealed that the inhibition of LRPPRC either by shikonin or by genetic knockdown resulted in a loss of LRPPRC expression (Figure 4I,J). Our data also showed that a 5 μM dose of shikonin triggered apoptosis (high expression of cleaved PARP, Figure 4I,J) only in SDHA- and LRPPRC-overexpressing cell lines, but not those with SDHA-KD or LRPPRC-KD. This suggests that SDHA-overexpressing cancer cells are particularly vulnerable to the LRPPRC inhibition, which leads to tumor cell death. Next, we examined the effect of shikonin on OXPHOS by measuring basal and maximal mitochondrial respiration (reflected as the oxygen consumption rate, OCR) in isogenic and non-related cell lines with and without LRPPRC-KD. We observed that in the SDHA- and LRPPRC-overexpressing cell line (BPPNM), both 1 μM and 5 μM shikonin concentrations most potently reduced mitochondrial respiration when compared to the isogenic LRPPRC-KD cell line or non-related SPCA cells (Supplementary Figure S9H,I). Interestingly, in BPPNM-LRPPRC-KD or SPCA cells, shikonin actually increased basal OCR at certain doses (Supplementary Figure S9I), which is likely due to mitochondrial hyperfusion, a compensatory mechanism maintaining mitochondrial respiration and energy production during cellular stress [39].

### 3.8. Shikonin Shows Potent Anti-Tumor Efficacy In Vivo in Ovarian Tumors with Concomitant Overexpression of SDHA and LRPPRC

**Figure 5:**
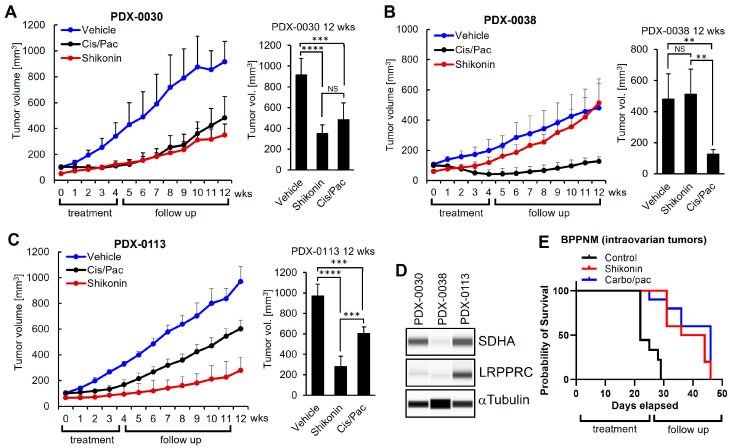
Evaluation of shikonin efficacy vs. chemotherapy in ovarian tumor models expressing SDHA or SDHA and LRPPRC. (**A**) Graph (left) represents high-SDHA, low-LRPPRC PDX-0030 tumor growth rate in NRG mice. Animals were treated for 4 weeks with vehicle, shikonin, or cisplatin/paclitaxel chemotherapy and follow-up for an additional 8 weeks to investigate the long-term effect of the treatment. Graph (right) shows differences in average tumor volume between treatment groups at 12 weeks since treatment started. Shikonin demonstrated similar anti-tumor efficacy to chemotherapy. (**B**) Graph (left) represents low-SDHA, low-LRPPRC PDX-0038 tumor growth rate in NRG mice treated as in (**A**). Graph (right) shows differences in average tumor volume between treatment groups at 12 weeks. In comparison to chemotherapy, shikonin lacks notable anti-tumor activity. (**C**) Graph (left) represents high-SDHA, high-LRPPRC PDX-0113 tumor growth rate in NRG mice treated as in (**A**). Graph (right) shows differences in average tumor volume between treatment groups at 12 weeks. Compared to chemotherapy, shikonin demonstrated superior treatment efficacy. (**A**–**C**) Asterisks indicate level of statistical significance: ** *p* ≤ 0.01, *** *p* ≤ 0.001, **** *p* ≤ 0.001, NS—not significant when assessed using one-way Anova test. (**D**) WES analysis of ovarian PDXs assayed for SDHA, LRPPRC, and loading control αTubulin. (**E**) Kaplan–Meier curves showing % probability of survival of C57BL/6J female mice injected into ovary with 5 × 10^5^ SDHA- and LRPPRC-overexpressing BPPNM tumor cells. Animals with established tumors started 4-week treatment with vehicle, shikonin, or chemotherapy and were monitored for tumor growth, metastasis, and survival (Kaplan–Meier method). Both shikonin and chemotherapy significantly prolonged mouse survival compared to the vehicle-treated group. Additional information is provided in Supplementary Figure S9.

Potent shikonin efficacy in vitro has been previously reported in ovarian cancer with upregulated SDHA [13]; however, there is a lack of respective in vivo data validating shikonin as affecting treatment in these tumors. In addition, the impact of LRPPRC overexpression on shikonin’s treatment efficacy has never been investigated. Here, we tested the efficacy of shikonin in vivo using clinically relevant patient-derived models of ovarian cancer. We selected two SDHA-overexpressing PDXs, one with low LRPPRC levels (PDX-0030), and the second with high LRPPRC levels (PDX-0113). We also included PDX with naturally low SDHA and LRPPRC levels (PDX-0038), as shown in Figure 5A–D and Supplementary Figure S9A–D [22]. Viable PDX fragments were implanted subcutaneously into the left flank of NRG female mice. When the tumor volume reached ~100 mm^3^, the mice started treatment. Animals were treated with vehicle control, shikonin (10 mg/kg, 3×/week), or cisplatin (5 mg/kg, 1×/week) + paclitaxel (10 mg/kg 1×/week). The treatment continued for 4 weeks, followed by an 8-week follow-up period to assess tumor growth and response to treatment or eventual disease relapse. During a 4-week treatment period, all treatment regimens significantly inhibited tumor growth when compared to the control group, and there was no difference in anti-tumor efficacy between shikonin and conventional chemotherapy (Supplementary Figure S9B–D). To assess the long-term efficacy of each treatment modality, we monitored the residual tumors for 8 weeks following discontinuation of all treatments. At the end of the follow up period, we observed that shikonin showed superior efficacy to chemotherapy in SDHA- and LRPPRC-overexpressing PDX-0113 (Figure 5C) and a similar efficacy to chemotherapy in high-SDHA and low-LRPPRC PDX-0030 (Figure 5A). In contrast, in the low-SDHA, low-LRPPRC PDX-0038, shikonin showed no therapeutic effect, while chemotherapy exhibited good efficacy (Figure 5B).

Furthermore, to determine shikonin’s antitumor efficacy in vivo in an immunocompetent mouse model of ovarian cancer, we used a high-SDHA, high-LRPPRC BPPNM tumor model implanted into the ovaries of C57BL/6J female mice. When tumors became palpable (tumor size ~100 mm^3^), the mice started a 4-week treatment with vehicle control, shikonin (10 mg/kg, 3×/week), or carboplatin (50 mg/kg, 1×/week) + paclitaxel (10 mg/kg 1×/week). The animals were monitored for tumor, metastases, and ascites development via palpation and caliper measurement. When mice reached advanced tumor burden, the animals were euthanized, and metastatic potential and overall survival were evaluated and compared between the groups. At necropsy, all mice developed metastatic tumors and ascites. Kaplan–Meier analysis of overall survival demonstrated that both shikonin and chemotherapy significantly improved mouse survival relative to the vehicle control, with each treatment extending the median survival time approximately twofold (Figure 5E, Supplementary Figure S9E–G).

Collectively, our in vivo data demonstrated that shikonin treatment results in a durable therapeutic response and can be an effective treatment in preselected SDHA- and LRPPRC-overexpressing ovarian tumors, which introduces hope for improving current therapies for ovarian cancer patients.

## 4. Discussion

Metabolic reprogramming is a distinguishing feature of cancer cells that undergo significant metabolic rewiring to adapt to increased energy demands to maintain rapid cell proliferation, promote metastasis, and survive microenvironmental stressors. However, cancer-specific metabolic adaptations also introduce distinct metabolic dependencies that offer a unique opportunity for therapeutic targeting. In this study, we studied the unique metabolism of ovarian cancer overexpressing SDHA and identified key vulnerabilities of tumors with the SDHA-gain-of-function phenotype that can be exploited for therapeutic benefit. While the role of succinate dehydrogenase (SDH) has been primarily studied in the context of its deficiency due to genetic mutations that can lead to neurodevelopmental disorders or rare tumors [40,41,42,43,44], the impact of aberrantly upregulated SDH in carcinogenesis is an emerging area of research [13,45,46].

Using human and mouse cell-line-based ovarian cancer models, we demonstrated that SDHA overexpression is associated with improved colony formation and cell survival in 3D suspension cultures but has no effect on cell proliferation in adherent 2D cultures (grown on plastic dishes), which is in agreement with our previous data [13]. Our findings reflect previous observations that the behavior of tumor cells in adherent 2D cultures does not fully predict the behavior of the corresponding tumor cells in 3D suspension cultures or in vivo systems regarding tumor-promoting functions or response to treatment [47,48,49]. Consistently, our in vivo study showed that the SDHA-overexpressing mFTE tumors exhibited significantly increased tumor growth and reduced mouse survival due to metastatic burden when compared with isogenic tumors with SDHA knockdown or unrelated tumors with low SDHA expression. Yong et al. reported similar results in breast cancer, showing improved proliferation, colony-forming, and migration capabilities of SDHA-overexpressing tumor cells vs. controls in suspension cultures in vitro [46]. Others reported that the high expression of SDHA was associated with a poor prognosis in breast cancer patients, an increased risk of metastasis and poor clinical outcome in uveal melanoma patients, and a shorter overall (OS) survival in head and neck cancer, while the SDHA deficiency in PDX models was linked with reduced tumor growth [46,50,51]. Studies exploring the role of other SDH subunits in cancer showed that patients with higher expression of SDHB displayed a worse OS than patients with reduced SDHB expression in renal carcinoma [52]. By contrast, patients with low SDHB expression presented a significantly shorter OS than those with high SDHB expression in nasopharyngeal carcinoma [53]. In a study by Li et al., the downregulation of all four SDH subunits (SDHA/B/C/D) correlated with a poor prognosis of hepatocellular carcinoma patients, which was associated with the accumulation of succinate acting as an oncometabolite in this tumor type [54]. Altogether, these findings suggest that the tumor-specific expression patterns of SDH subunits and their prognostic significance may vary depending on the tumor type. Nevertheless, in ovarian and breast cancer, SDHA subunit overexpression promotes an aggressive tumor phenotype characterized by an improved ability to survive, proliferate, and metastasize in anchorage-independent conditions. These cancer features are particularly relevant in ovarian cancer disease, where tumor cells are able to thrive and robustly disseminate through the ascitic fluid in the peritoneal cavity.

In this study, the primary finding from the proteomic and metabolomic analyses revealed that the TCA cycle pathway plays an important role in ovarian tumors with the SDHA-gain-of-function phenotype. We demonstrated that the TCA cycle protein content progressively increased from hFTs to PDXs, reaching the highest abundance in SDHA-overexpressing PDXs compared to PDXs with low/normal SDHA expression. Consistent with our observations, it has been shown that increased mitochondrial activity, specifically the overactive TCA cycle, plays a significant role in providing metabolic advantages to ovarian cancer cells, promoting tumor progression, and maintaining cancer stem cell-like behavior and chemotherapy resistance [7,11,55]. Furthermore, by employing HRA-MS and stable-isotope tracing, we discovered that tumor cells with SDHA overexpression show elevated levels of αKGDH complex and increased glutamine-derived carbon flux into the TCA cycle when compared to controls. It has been shown that the αKGDH complex collaborates with glutaminolysis, governing αKG flux through the TCA cycle [36,37,38]. Within the TCA cycle, the αKG can undergo either reductive carboxylation to support fatty acid biosynthesis or oxidative decarboxylation to generate reduced electron carriers such as NADH and FADH_2_ to support energy production via OXPHOS [29]. Similarly to our findings, Udumula et al. demonstrated that highly metabolically active ovarian cancer cell lines have significantly increased mRNA and protein expression of DLST (a subunit of αKGDH), a key enzyme facilitating glutamine entry into the TCA cycle [55]. Furthermore, Yang et al., using isotope tracing, showed that highly invasive ovarian cancer cells are heavily reliant on glutamine to maintain their TCA cycle metabolite pool and to support cell proliferation and invasiveness, whereas low-invasiveness tumors showed glutamine-independent growth [12]. In addition, the authors also found that genes involved in glutaminolysis and the TCA cycle pathways were highly expressed in ovarian cancer patients with poor survival, whereas glycolytic genes were associated with better patient survival [12]. Others showed that aggressive chemoresistant ovarian cancer cell lines exhibit increased metabolic activity, reflected as increased OXPHOS coupled with the TCA cycle, when compared to chemosensitive cell lines [7]. Interestingly, in many tumor types, the reductive carboxylation of glutamine-derived αKG promotes tumor growth by supplying citrate for de novo fatty acid synthesis [56,57]. However, here, we showed that the SDHA-overexpressing ovarian tumors rely on increased oxidative metabolism of αKG, promoting OXPHOS rather than de novo lipid biosynthesis. Our observations reflect ovarian cancer behavior in vivo, where advanced tumors preferentially depend on the uptake of exogenous lipids and cholesterol rather than de novo lipogenesis, especially in the presence of adipocytes [58]. Adipocytes act as major mediators of ovarian cancer metastasis to the fat-rich omentum tissue. Subsequently, adipocytes provide fatty acids to the cancer cells, fueling rapid tumor growth [58,59]. In light of these data, it is likely that the SDHA-overexpressing aggressive tumors use adipocyte-derived lipids for tumor growth while relying on glutaminolysis to replenish the TCA cycle with carbon intermediates essential for mitochondrial OXPHOS metabolism and biosynthesis pathways to support cancer progression and survival.

Our previous in vitro drug screening study [13] revealed that the most selective and potent compound in targeting SDHA-overexpressing cancer cells is shikonin; however, the underlying mechanism is unclear [15,16,17]. Here, we performed a mass spectrometry-based drug target discovery study and identified LRPPRC as a key binding partner of shikonin. LRPPRC is a versatile protein with an essential role in regulating mitochondria homeostasis and energy metabolism [18,19]. LRPPRC functions as a mitochondrial mRNA chaperone regulating the expression of mitochondrial genes, particularly respiratory chain complexes [18,60]. In recent years, overexpressed LRPPRC emerged as a potent tumor oncogene linked with poor patient prognosis in various human cancers [61,62,63,64,65]. LRPPRC is implicated in driving cancer progression by integrating signals from upstream regulators, engaging with interaction partners, and modulating downstream targets [66]. Our novel findings shed light on an unexplored link between SDHA and LRPPRC in ovarian cancer, and to the best of our knowledge, there are no studies directly investigating a functional relationship between those key metabolic regulators. Therefore, we analyzed GeoMx spatial transcriptomics data to determine the SDHA and LRPPRC expression patterns in human HGSOC samples to provide insight into their potential roles in driving ovarian cancer onset and disease progression. We observed a progressive increase in SDHA and LRPPRC transcript expression in early stages of ovarian carcinogenesis, which subsequently led to a concomitant high expression of SDHA and LRPPRC gene and protein in advanced tumors. Genomic studies show that the mechanism of SDHA overexpression is mainly due to SDHA gene amplification, which is particularly common in HGSOC [13]. The SDHA gene resides within the 5p15 chromosome locus, which is one of the most significantly amplified regions in the HGSOC genome [67]. HGSOC tumors are characterized by a high degree of somatic copy number aberrations (SCNAs), largely caused by a high prevalence of mutations and promoter methylation in DNA repair genes, which contribute to extensive chromosomal instability [67]. In other tumor types, the upregulation of the SDHA gene was driven by different mechanisms, including SDHA promoter hypomethylation in breast cancer [46] or increased histone acetylation within the SDHA promoter in multiple myeloma [68]. The LRPPRC gene amplification is relatively rare in HGSOC, since the LRPPRC gene (chromosome 2p21 locus) is not located in genome regions affected by recurrent focal amplification or deletion events [67]. There is a scarcity of data regarding the mechanisms contributing to LRPPRC overexpression in tumors. Xue et al. showed that reduced methylation of the LRPPRC promoter correlates with an increased DNA copy number of the LRPPRC gene and subsequently increased LRPPRC expression in breast cancer [69]. It is possible that a similar mechanism occurs in other tumor types, including HGSOC.

Further, we showed that the inhibition of LRPPRC either by shikonin or by genetic knockdown (KD) resulted in a loss of LRPPRC expression, while SDHA levels remained unchanged. These observations agree with previous studies investigating the effects of LRPPRC deficiency on the activity and assembly of ETC complexes. Cuillerier et al. showed that the hepatocyte-specific inactivation of LRPPRC in mice leads to a severe decrease in activity and assembly of Complex IV (CIV) and CV, while the amounts of assembled CI, CII, and CIII and the TCA cycle enzymes’ activity were similar to controls, including normal levels of SDHA [70]. In patients with French-Canadian Leigh Syndrome (LSFC), which is an autosomal recessive OXPHOS disorder caused by a mutation of the LRPPRC gene, the assembly of all ETC complexes is disrupted, with the exception of the nuclear-encoded subunits of CII and CV, which results in normal SDHA levels in patients. These data are consistent with our findings showing that loss of LRPPRC does not affect SDHA levels but is associated with OXPHOS deficiency [71]. In contrast to LRPPRC KD, which only downregulated LRPPRC expression, the SDHA KD significantly reduced the expression of both SDHA and LRPPRC. These findings highlight the possibility of the upregulated SDHA acting as a positive regulator of LRPPRC in ovarian cancer, which is yet an unexplored area of research. Since LRPPRC gene expression could be regulated by altered promoter methylation, it would be interesting to investigate if the TCA cycle metabolites, particularly those associated with the SDHA overexpression phenotype (increased fumarate levels [13], Figure 3E), are able to indirectly affect LRPPRC expression via epigenetic mechanisms [72].

Our Seahorse-based assays showed that LRPPRC plays an essential role in promoting OXPHOS in SDHA-overexpressing ovarian cancer cell lines. The inhibition of LRPPRC by shikonin in those cell lines significantly suppressed mitochondrial respiration, leading to bioenergetic dysfunction and cancer cell death. Interestingly, while cell lines with high expression of SDHA and LRPPRC showed severe inhibition of mitochondrial respiration resulting in apoptosis upon treatment with shikonin, the cell lines with LRPPRC KD or naturally low LRPPRC levels were completely insensitive to low doses of shikonin, which is likely due to mitochondrial hyperfusion. Mitochondrial hyperfusion is a widely conserved pro-survival mechanism to safeguard ETC performance and mitochondrial integrity during periods of OXPHOS impairment in response to various stressors [39,73]. It is likely that normal cells or cancer cells with normal SDHA levels are able to preserve mitochondrial integrity and the ETC function by employing mitochondrial hyperfusion. In contrast, it seems that in tumors with high-energy metabolism, such as those with SDHA overexpression, mitochondrial hyperfusion could be defective or insufficient to maintain energy production via OXPHOS, resulting in cell death [39]. This also emphasizes the idea that LRPPRC inhibitors could be less toxic to normal cells while eliminating SDHA/LRPPRC-overexpressing cancer cells. Future mechanistic studies investigating the impact of mitochondrial hyperfusion on cell viability in SDHA-overexpressing tumors treated with LRPPRC inhibitors are warranted.

## 5. Conclusions

In summary, we investigated the unique metabolic features of ovarian cancer and discovered that tumors with a high-OXPHOS phenotype are characterized by the concomitant overexpression of SDHA and LRPPRC. We demonstrated for the first time that the SDHA and LPPPRC expression gradually increases during the transition from early precancerous lesions to late-stage metastatic HGSOC, becoming significantly upregulated in approximately 20% of advanced tumors. Importantly, our in vivo findings demonstrated that inhibition of LRPPRC function by shikonin results in a sustained therapeutic benefit, as evidenced by a two-fold extension of median survival in mouse models of ovarian cancer. These results suggest that shikonin could be an effective therapy in preselected SDHA- and LRPPRC-overexpressing ovarian tumors. Consequently, we propose that high levels of SDHA and LRPPRC could serve as novel biomarkers of high-OXPHOS tumors to guide personalized cancer therapy.

## Data Availability

The data presented in this study are contained within the article or the Supplementary Materials. Additional data supporting the findings of this study are available from the corresponding author upon reasonable request.

## Supplementary Materials

The following supporting information can be downloaded at: https://www.mdpi.com/article/10.3390/cancers17121942/s1, Figure S1: A comprehensive description of materials and experimental procedures. Figure S2: The effect of SDHA overexpression on cell proliferation and anchorage-independent growth. Figure S3: An in vivo study evaluating the effect of SDHA overexpression on tumor growth and mouse survival. Figure S4: An analysis of protein abundance in metabolic pathways assessed using HRA-MS. Figure S5: The effects of SDHA overexpression on glucose and glutamine flux in ovarian cancer. Figure S6: The identification of LRPPRC protein as a top molecular target of shikonin. Figure S7: The analysis of the direct binding of shikonin to the LRPPRC protein. Figure S8: *SDHA* and *LRPPRC* gene expression during HGSOC development and progression. Figure S9: The effect of shikonin on OXPHOS metabolism and tumor growth in ovarian cancer models with and without SDHA and/or LRPPRC overexpression.

## Abbreviations

The following abbreviations are used in this manuscript:

2D	two-dimensional.
3D	three-dimensional.
3-PGA	3-phosphoglyceric acid.
αKG	αketoglutarate.
αKGDH	αketoglutarate dehydrogenase.
ACAA2	acetyl-CoA acyltransferase 2.
ACADL	acyl-CoA Dehydrogenase Long Chain.
ANOVA	analysis of variance.
ATCC	American type culture collection.
ATP	adenosine triphosphate.
BSA	bovine serum albumin.
CAT	catalase.
CI	complex I.
CII	complex II.
CIII	complex II.
CIV	complex IV.
CV	complex V.
CS	citrate synthase.
DHAP	dihydroxyacetone phosphate.
DLST	dihydrolipoamide S-succinyltransferase.
DOX	doxycycline.
ECAR	extracellular acidification rate.
ES6B	epoxy-activated Sepharose 6B.
ETC	electron transport chain.
F1,6BP	fructose-1,6-bisphosphate.
F6P	fructose 6-phosphate.
FADH2	flavin adenine dinucleotide.
FCCP	carbonyl cyanide-4-(trifluoromethoxy)phenylhydrazone.
FT	fallopian tube.
G6P	glucose-6 phosphate.
GAPDH	glyceraldehyde 3-phosphate dehydrogenase.
GLU	glucose.
GLUD1	glutamate dehydrogenase 1.
GLN	glutamine.
hFT	human fallopian tube.
HGSOC	high-grade serous ovarian cancer.
HK2	hexokinase 2.
H	hour.
HPLC	high-performance liquid chromatography.
HRA-MS	high-resolution accurate mass spectrometry.
IACUC	institutional animal care and use committee.
IDH3A	isocitrate dehydrogenase (NAD(+)) 3 catalytic subunit alpha.
IDH3B	isocitrate dehydrogenase (NAD(+)) 3 catalytic subunit beta.
IRB	institutional review board.
KD	knockdown.
LC-MS-MS	liquid chromatography–tandem mass spectrometry.
LC-Q/TOF	liquid chromatography–quadropole/time-of-flight.
LRPPRC	leucine-rich pentatricopeptide repeat-containing.
LSFC	French-Canadian Leigh Syndrome.
mFT	mouse fallopian tube.
mFTE	murine fallopian tube epithelium.
MS	mass spectrometry.
MTT	3-(4,5-Dimethylthiazol-2-yl)-2,5-Diphenyltetrazolium Bromide.
NADH	nicotinamide adenine dinucleotide hydrogen.
OAA	oxaloacetate.
OCR	oxygen consumption rate.
OGDH	oxoglutarate dehydrogenase.
OE	overexpression.
OMRF	Oklahoma Medical Research Foundation.
OS	overall survival.
OXPHOS	oxidative phosphorylation.
PDX	patient-derived xenograft.
PEP	phosphoenolpyruvate.
PRDX2	peroxiredoxin 2.
PPP	pentose phosphate pathway.
RT	room temperature.
SCNA	somatic copy number aberration.
SD	standard deviation.
SDH	succinate dehydrogenase.
SDHA	succinate dehydrogenase subunit A.
SDS-PAGE	sodium dodecyl-sulfate polyacrylamide gel electrophoresis.
SEM	standard error of the mean.
shRNA	short hairpin ribonucleic acid.
SLC25A20	solute carrier family 25 member 20.
SUCLG1	succinate-CoA ligase GDP/ADP-forming subunit alpha.
STIC	serous tubal intraepithelial carcinomas.
STR	short tandem repeat.
TCA	tricarboxylic acid.
TME	tumor microenvironment.
USG	ultroser G serum substitute.
