# Supplementary material for: The Identification of a Key Regulator of Mitochondrial Metabolism, the LRPPRC Protein, as a Novel Therapeutic Target in SDHA-Overexpressing Ovarian Tumors
Source: Cancers (Basel). 2025 Jun 11;17(12):1942. doi: 10.3390/cancers17121942 (PMC12190274; doi:10.3390/cancers17121942)
Supplement: Supplementary file 1 [file cancers-17-01942-s001.zip › Supplementary Figure S9.pdf]

WES analysis of SDHA and LRPPRC levels in selected ovarian PDX models

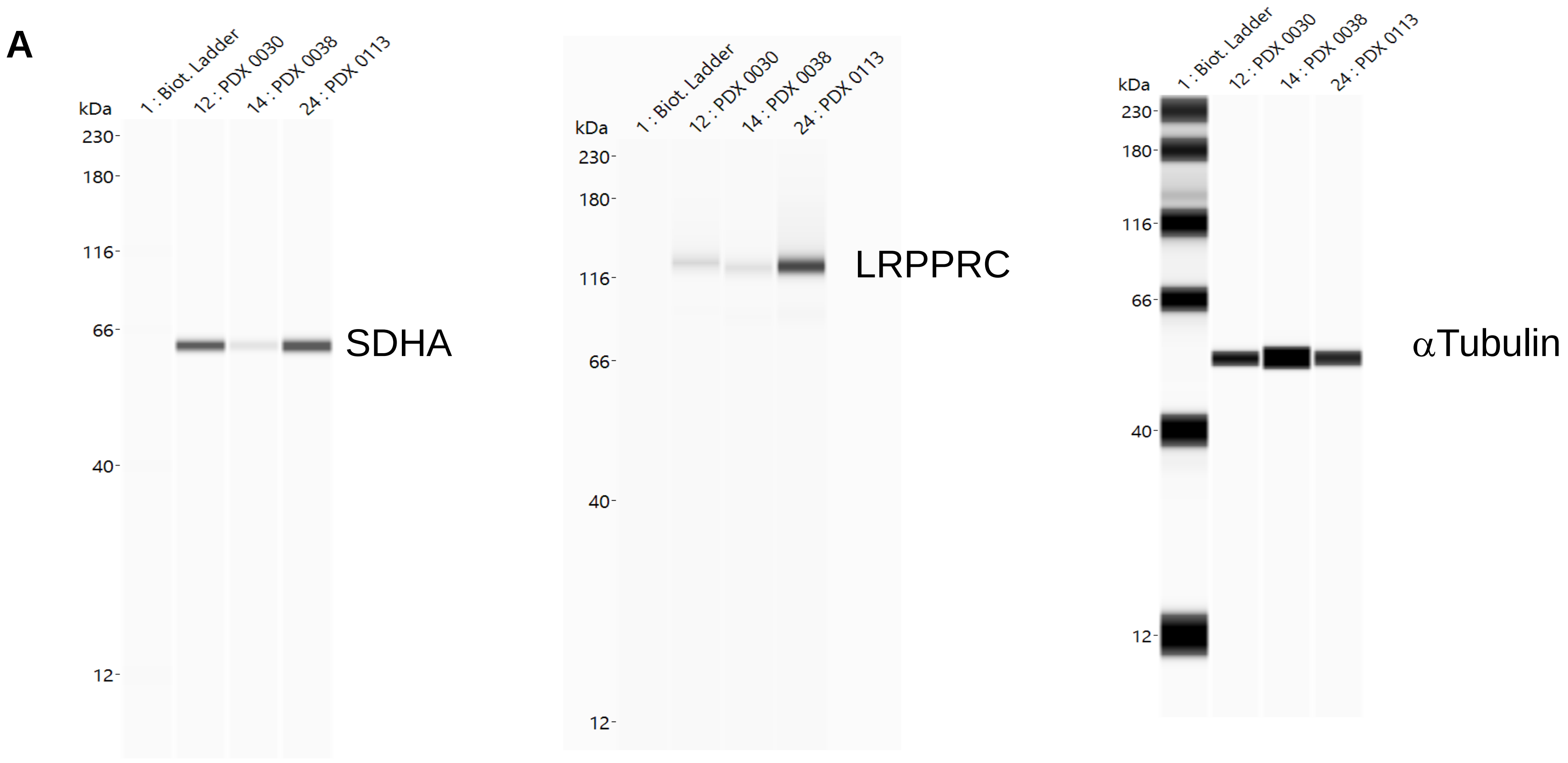

Supplementary Figure S9. (A) Uncropped WES images showing the expression of SDHA, LRPPRC and αTubulin (loading control) in selected ovarian PDX models.

The effect of shikonin or chemotherapy in ovarian PDXs with and without SDHA or LRPPRC overexpression

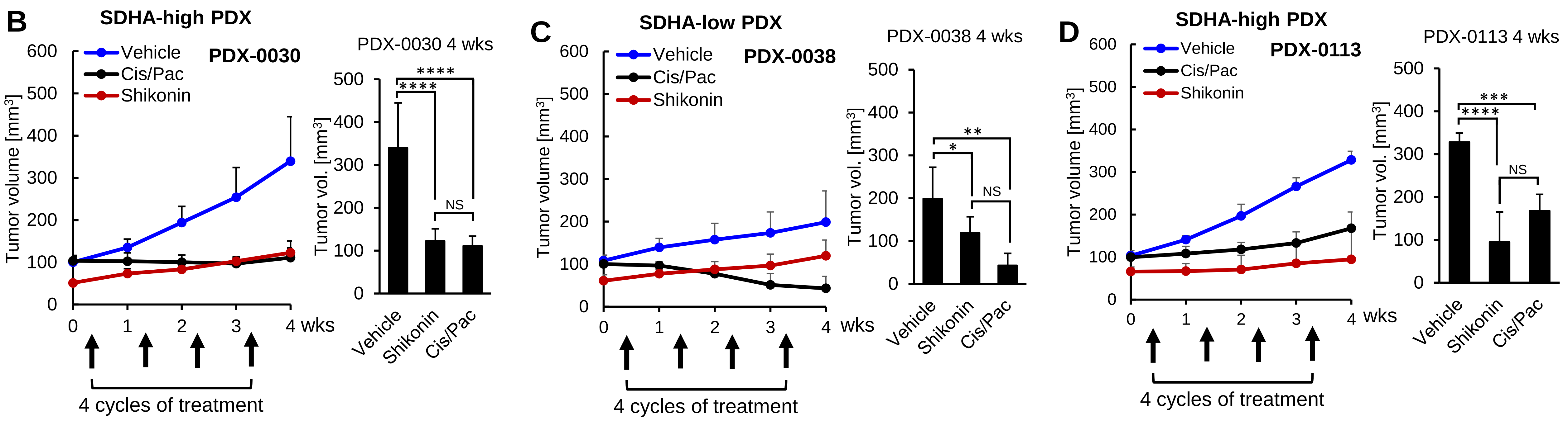

Supplementary Figure S9. (B) Graph (left) represents SDHA-high, LRPPRC-low PDX-0030 growth rate during therapy with 4 cycles of treatment including vehicle control (PBS), shikonin (10 mg/kg, 3x/week) or chemotherapy (carboplatin 50 mg/kg, 1x/week and paclitaxel, 10 mg/kg 1x/week). Graph (right) shows differences in average tumor volume between mice groups after 4 weeks of treatment. **(C)** Graph (left) represents SDHA-low, LRPPRC-low PDX-0038 growth rate during therapy with 4 cycles of treatment as in 'B'. Graph (right) shows differences in average tumor volume between mice groups after 4 weeks of treatment. **(D)** Graph (left) represents SDHA-high, LRPPRC-high PDX-0113 growth rate during therapy with 4 cycles of treatment as in 'B'. Graph (right) shows differences in average tumor volume between mice groups after 4 weeks of treatment. **(B-D)** Statistical significance of data were evaluated by one-way Anova. Asterisks indicate level of statistical significance: * P ≤ 0.01, ** P ≤ 0.01, ***P ≤ 0.001, ****P ≤ 0.001, NS not significant

Survival of mice implanted orthotopically (into ovary) with BPPNM tumor model endogenously overexpressing SDHA and LRPPRC (BPPNM) treated with shikonin and carboplatin/paclitaxel vs. untreated control

E

BPPNM - Control										
**Mouse number**	M1	M2	M3	M4	M5	M6	M7	M8	M9	
**Tumor weight (g)**	3.4	2.3	3.3	1.8	2.9	3.4	3.2	3.1	1.9	
**Resected tumor**	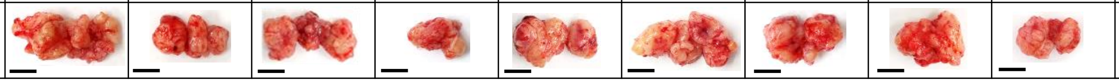									

BPPNM - Shikonin										
**Mouse number**	M11	M12	M13	M14	M15	M16	M17	M18	M20	M31
**Tumor weight (g)**	2.2	3.1	1.7	2.0	1.6	1.0	2.8	1.5	1.1	2.3
**Resected tumor**	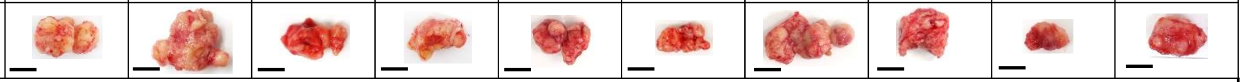									

BPPNM – Carboplatin/Paclitaxel										
**Mouse number**	M21	M22	M23	M25	M26	M27	M28	M29	M30	M33
**Tumor weight (g)**	1.8	3.2	0.9	3.9	1.8	2.9	1.4	2.6	2.5	1.9
**Resected tumor**	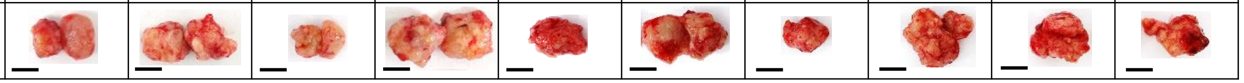									

F

Control

Shikonin

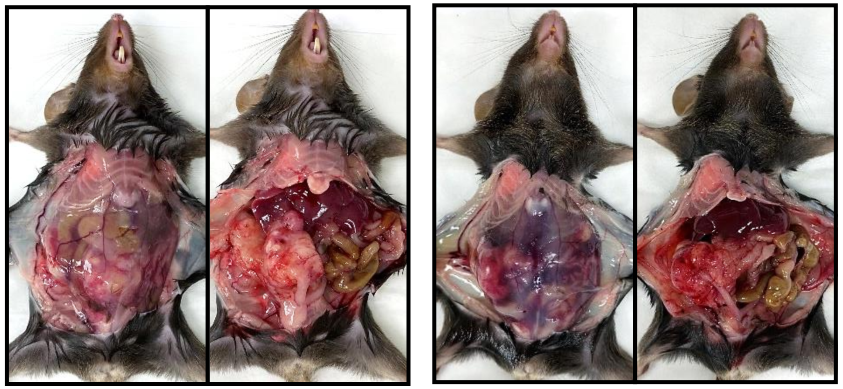

Carbo/Pac

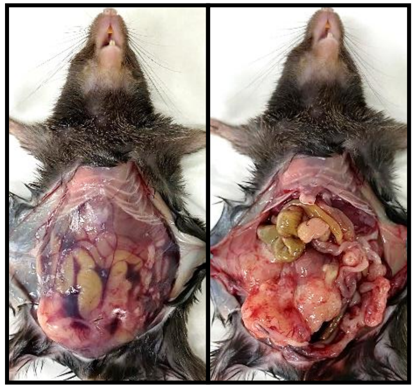

G

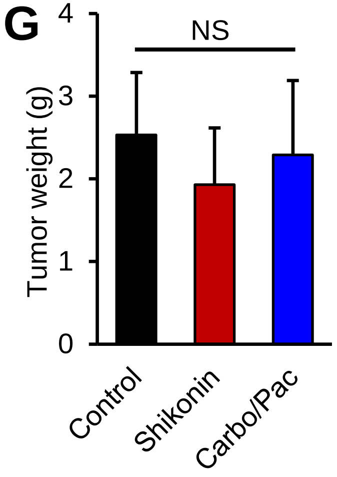

Supplementary Figure S9. (E) Intraovarian tumors harvested from mice described in Figure 5E in the main manuscript. The tumors were harvested at endpoint of the experiment when mice became moribund. **(F)** Representative images of ascites and metastases in C57BL/6J female mice implanted into ovary with $3x10^6$ BPPNM tumor cells followed by treatment with vehicle control, shikonin or chemotherapy (carboplatin/paclitaxel). **(G)** Graph shows average weight of tumors shown in the table 'E'. Statistical significance of data were evaluated by one-way Anova, NS not significant.

The effect of shikonin on mitochondrial respiration in mFTE cell lines

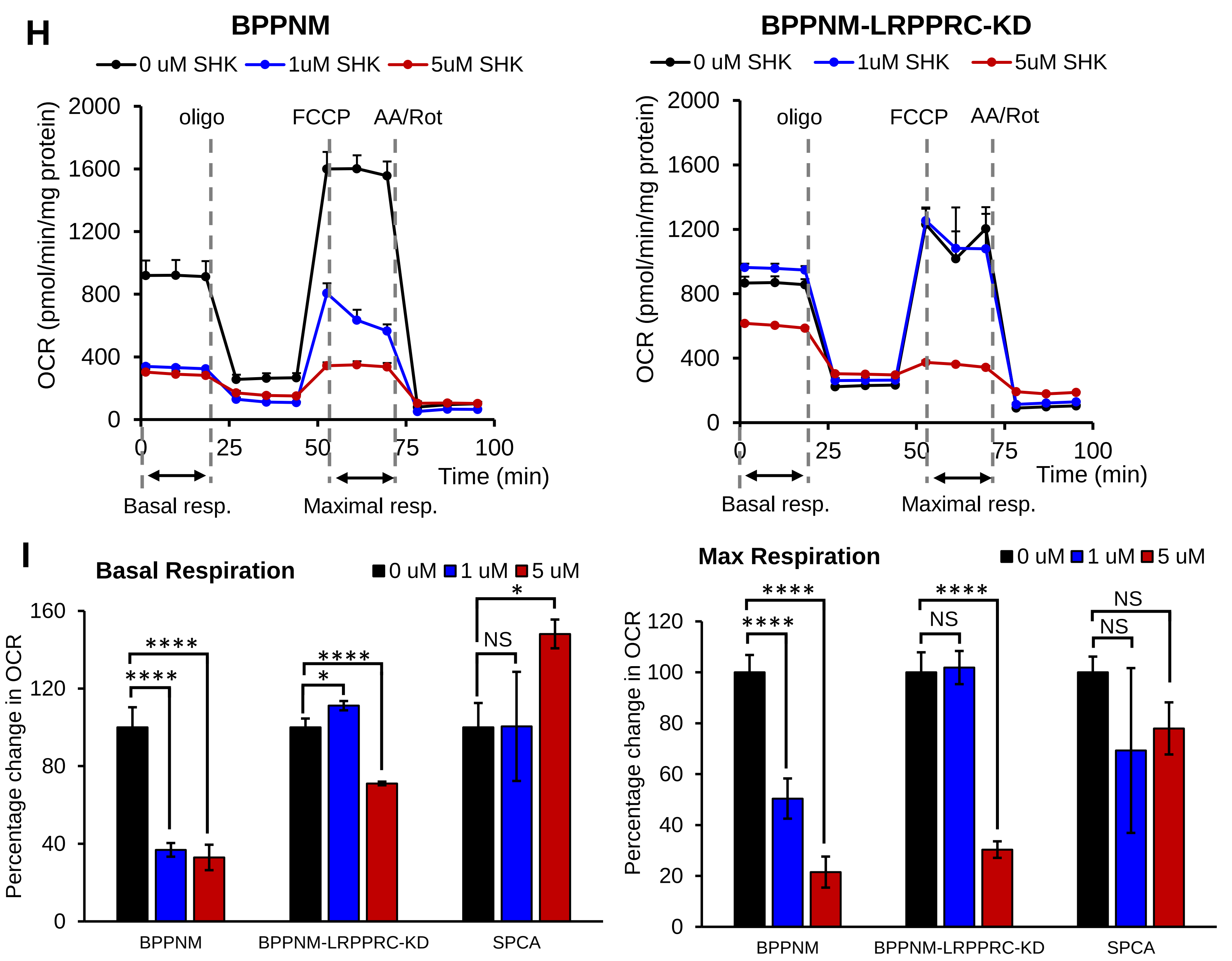

Supplementary Figure S9. (H) OCR was measured in mFTE cell lines with and without SDHA overexpression or LRPPRC-KD by the Seahorse XF Cell Mito Stress Test. Cells were challenged by oligomycin A “oligo”, FCCP, and antimycin A + rotenone “AA/Rot **(I)** Graphs represent percentage change of basal or maximal respiration in mFTE cell lines shown in ‘A’ following shikonin treatment (one-way Anova). Asterisks indicate level of statistical significance: * P ≤ 0.01, ****P ≤ 0.001, NS not significant.
