# Supplementary material for: The Identification of a Key Regulator of Mitochondrial Metabolism, the LRPPRC Protein, as a Novel Therapeutic Target in SDHA-Overexpressing Ovarian Tumors
Source: Cancers (Basel). 2025 Jun 11;17(12):1942. doi: 10.3390/cancers17121942 (PMC12190274; doi:10.3390/cancers17121942)
Supplement: Supplementary file 1 [file cancers-17-01942-s001.zip › New Supp Figure S1 - SDHA levels -Materilas Methods 6-5-25.pdf]

Materials and Methods (Supplementary information)

A

Maintenance and validation of ovarian cancer cell lines

Human ovarian cancer cell lines were maintained in RPMI 1640 Gibco^{TM} medium (#11875-093), Thermo Fisher Scientific, Waltham, MA, USA) supplemented with 10% fetal bovine serum (FBS) (#F0926, Sigma-Aldrich, St. Louis, MO, USA). FT190 and FT194 cell lines were maintained in DMEM/Hams F-12 50/50, medium (#10-090-CV, Corning, Glendale, AR, USA) supplemented with 2% USG (Ultroser G Serum Substitute) serum (#67042, Crescent Chemicals, Islandia, NY, USA). The mFTE cell lines were maintained in DMEM, high glucose, HEPES Gibco^{TM} medium (#12430054) supplemented with 10 mg/ml EGF, Gibco^{TM} (#AF-100-15-1MG), Insulin-Transferrin-Selenium (ITS-G), Gibco^{TM} (#41400045), and 5% FBS. All cell lines were maintained in a standard humidified incubator at 37 °C in 5% CO_2 and 95% O_2 atmosphere. To ensure quality of data and to avoid issues associated with cell line misidentification, contamination or genetic drift, the cell lines were purchased from validated reliable source and cryopreserved in the laboratory cell line bank at low passage (passage 1–3). In addition, cell lines were tested for Mycoplasma by Idexx BioAnalytics and were found negative for any contamination. OVCAR3, 4, and 8 cell lines were authenticated by short tandem repeat (STR) profiling by ATCC. It is important to acknowledge the potential limitations associated with the use of newly established cell lines, such as mouse ovarian cancer cell lines derived from murine fallopian tube epithelium (mFTE). Newly derived cell lines may better reflect tumor-specific or early-stage disease characteristics, which might not align with features of extensively passaged standard cell lines, often derived from late-stage or treatment-resistant cancers; they often lack extensive molecular and functional characterization potentially affecting reproducibility; and variability in passage number and time since derivation may introduce biological differences that complicate direct comparisons with well-established cell lines.

Quantification of protein abundance using High Resolution Accurate Mass Spectrometry (HRA-MS) Human fallopian tube and PDX tissues were minced and lysed in Buffer B and 100 µg of protein was used for analysis. Bovine serum albumin (BSA) was added as a non-endogenous internal standard. The samples were mixed, heated at 70°C, and precipitated with 80% acetone at −20°C overnight. The protein precipitate was reconstituted in Laemmli sample buffer at 1 ug/µL and separated using SDS-PAGE. The gel was fixed and stained with GelCode Blue, and the 1.5-cm lane was cut out of the gel. Gel pieces were washed, reduced with DTT, alkylated with iodoacetamide, and digested with trypsin at room temperature (RT) overnight. Digested peptides were extracted with 50% methanol and 10% formic acid. The extracts were evaporated and dissolved in 1% acetic acid for analysis. Protein abundance was determined using the HRA-MS method, scanning m/z 300–1100 with a resolution of 280,000, with an orbitrap mass spectrometer (Thermo Scientific, QEx Plus) configured with a High-Performance Liquid Chromatography (HPLC) system (Thermo Scientific Ultimate 3000). The HPLC conditions were a linear gradient elution from 2% B to 45% B in 60 min. Absolute protein concentrations were determined using multiple validated peptide markers to determine abundance of each protein. Protein abundance was determined by normalization to BSA used as a non-endogenous internal standard. Housekeeping proteins were also used for normalization. Further, the data were processed using Skyline version 3.7.0.10940. The program finds and integrates the proper chromatographic peaks. Proper retention times are predicted based on retention time calibration using BSA and trypsin peptides. This process is edited by manually inspecting the data as needed. Assays are set to find and integrate 2 peptides per protein. All peptides used have been validated in prior experiments. Calculations determine the total protein response from the geomean of the two monitored peptides. Results are normalized to the BSA internal standard and expressed as pmol/100µg total protein.

Materials and Methods (Supplementary information)

B

Metabolic flux of stable isotope labelled [U^{13}C]-glucose and [U^{13}C]-glutamine

Briefly, ^{13}C metabolic flux analysis (^{13}C-MFA) was performed using uniformly labeled [U^{13}C]-glucose (#CLM-1396) and [U^{13}C]-glutamine (#CLM-1822) from Cambridge Isotope Laboratories in SDHA overexpressing ovarian cancer cells (OVCAR4-SDHA) vs. controls (OVCAR4). Doxycycline was added 24 hours prior to the metabolic flux experiment to induce overexpression of SDHA in OVCAR4 cells. On the day of the flux experiment, the RPMI media was replaced with Agilent Seahorse XF base media (glutamine and glucose free media) supplemented with 10 mM ^{13}C-labeled glucose and 2 mM of 13C-labeled glutamine, and cells were incubated for 30 minutes at 37 °C. Cells were then collected after two PBS washes and immediately snap frozen. Metabolites were then extracted with 8:2(v/v) methanol: water, followed by sonication and centrifugation. The supernatant was dried and reconstituted in 7:3 (v/v) acetonitrile: water and analyzed for carbon-13 isotopic enrichment in glycolytic and TCA cycle intermediates by liquid chromatography-quadrupole/time-of-Flight mass spectrometry (6546 LC/Q-TOF) coupled to 1290 Infinity II LC from Agilent Technologies, Santa Clara, CA, USA. Chromatographic separation was performed on an Agilent InfinityLab Poroshell 120 HILIC-Z, 2.1 × 100 mm, 2.7 μm (p/n 675775-924), with a UHPLC Guard, HILIC-Z, 2.1 mm × 5 mm, 2.7 μm (p/n 821725-947). Time course labelling was first conducted in OVCAR4 control cells to estimate dynamic and steady-state labelling timepoints. The abundance and flux of [U^{13}C]-glucose-derived and [U^{13}C]-glutamine-derived metabolic intermediates were assessed by tracking and quantification of isotopomers metabolized within respective metabolic pathways. Metabolite abundance and isotopic enrichments were calculated using Agilent Profinder software.

Shikonin was conjugated with epoxy-activated Sepharose 6B (ES6B)

ES6B incubated with vehicle only was used as negative control. Briefly, ES6B was washed in distilled water followed by washing in coupling buffer (1M NaCl, adjusted to pH 12 with 1M NaOH). ES6B medium was generated by resuspension of ES6B (1 g per 3.5 ml of coupling buffer). Shikonin (200 μmol per ml) or vehicle control was dissolved in coupling buffer, added to ES6B medium (1:1 ratio), and gentle mixed (16 h at 30°C) in a stopper vessel. Next day, an excess of ligand (shikonin) or vehicle was washed away using coupling buffer and any remaining active groups within ES6B were blocked with 1M ethanolamine pH 8 by overnight incubation at RT. Next, ES6B-shikonin and ES6B-vehicle conjugates were washed with 3 cycles of alternating pH using sintered glass filter system. Each cycle consisted of a wash with acidic wash buffer (0.1M acetate buffer containing 0.5M NaCl, pH 4) followed by a wash with alkaline wash buffer (0.1M Tris-HCl buffer containing 0.5M NaCl, pH 8). Finally, ES6B-shikonin and ES6B-vehicle were resuspended in binding buffer (0.05 M Tris-HCl, and 0.15 M NaCl, pH 7.5), and stored for the next step to be incubated with cell lysates. Respective cell line lysates were prepared in RIPA buffer (0.05M Tris-HCl, 1% NP-40, 0.25% sodium deoxycholate, 0.15M NaCl, 1 mM EDTA, 1 mM Na_3VO_4, and 1x protease inhibitor cocktail, pH 7.4). Lysates containing 1 mg of protein were mixed with 100 μl ES6B-shikonin or 100 μl ES6B-vehicle to a total volume of 400 μl. The mixture was incubated with gentle stirring for 16 h at 4°C and spun down for 2 min, 5,000 rpm at 4°C. The supernatant was discarded, and the precipitate was washed 3 times with 500 μl of chilled RIPA Buffer. Next, shikonin-bound or vehicle-bound proteins were eluted using denaturizing 2x SDS Buffer by adding 100 μL of 2x SDS Buffer to the pelleted precipitate, boiled for 10 min, and spun down (10 min at 10,000 rpm at 4°C). The supernatants containing shikonin-bound proteins, as well as those from vehicle control samples underwent a mass spectrometry analysis.

Materials and Methods (Supplementary information)

C

Measurement of OCR and ATP production rate by Seahorse

To perform the Seahorse XF Cell Mito Stress assay (#103015-100) assay, cells were evenly seeded (60,000 cells/well; following optimization of cell seeding number) into the XF24 cell culture plate (#102340-100, Seahorse XFe24 FluxPaks, Agilent Technologies) and incubated for 24 h at 37 °C and 5% CO_2. After 24 h of incubation, cell culture medium was replaced with Seahorse XF Base Medium (#103335-100, Agilent Technologies) supplemented with 2 mM L-Glutamine (#G7513-100ML, Sigma-Aldrich, St. Louis, MO, USA), 1 mM Sodium Pyruvate (#13-115E, Lonza Bioscience, Basel, Switzerland), 10 mM Glucose (#G8270-100G, Sigma-Aldrich) with pH adjusted to 7.4. Then, the cells were placed in a non-CO_2 incubator for 1 h at 37 °C required for cells to reach an optimal pH and temperature conditions prior to the start of the experiment. Following 1 h incubation, the XF24 cell culture plate was loaded into the Seahorse instrument, which measured OCR/ECAR at intervals of approximately 5–8 min. Depending on the seahorse assay, various pharmacological compounds interrupting mitochondrial respiration were injected via ports to determine their effects on mitochondria function. The plate included also control blank wells containing only media to which various reagents were added similar to experimental wells. The blanks were automatically subtracted from experimental wells by instrument software. Three measurements of OCR/ECAR were obtained following injection of each compound modulating cellular respiration. Compounds used in Seahorse assays included 1 mM Oligomycin A (ATP synthase inhibitor, #495455-10MG), 1 mM of FCCP (protonophoric uncoupler, #C2920-10MG), 0.5 mM of Antimycin A (complex III inhibitor, #A8674-25MG), and 0.5 mM of Rotenone (complex I inhibitor, #R8875-25G) from MilliporeSigma, Burlington, MA, USA. To determine the effect of shikonin on OCR, and ATP production, the cells were treated with indicated concentrations of shikonin for 1 h prior starting Cell Mito Stress assay. Compound concentrations were optimized prior to experiments. The measurements were normalized with cell number and total protein levels (Bradford protein assay).

Validation of mFTE cell lines harboring patient-relevant mutant genotypes

D

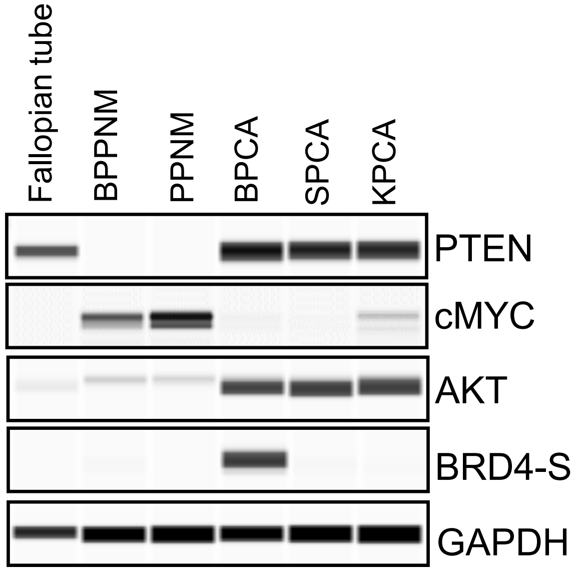

E

mFTE cell lines:

BPPNM (Brca1$^{-/-}$p53$^{-/-R172H}$Pten$^{-/-}$Nf1$^{-/-}$Myc^{OE})

PPNM (p53$^{-/-R172H}$Pten$^{-/-}$Nf1$^{-/-}$Myc^{OE})

BPCA (BRD4$^{short\ isoformOE}$p53$^{-/-R172H}$Ccne1^{OE}Akt2^{OE})

SPCA (Smarca4^{OE}p53$^{-/-R172H}$Ccne1^{OE}Akt2^{OE})

KPCA (KRAS^{G12V}p53$^{-/-R172H}$Ccne1^{OE}Akt2^{OE})

Supplementary Figure S1. Validation of engineered mFTE cell lines by Wes (ProteinSimple). (D) The respective genotypes of mFTE cell lines were verified by analysis of the expression of selected protein markers. WES images showing expression levels of PTEN, cMYC, AKT and BRD4-S in mFTE cell lines vs. normal murine fallopian tube cells, which reflects respective genetic profiles of each cell line (as shown in ‘B’). GAPDH expression was used as a loading control. Uncropped WES images are shown in S1C. (**E)** Genotypes of mFTE cell lines. These cell lines were developed by Dr. Weinberg group and described in the publication by Iyer et. al., Cancer Discov., 2021. *“Genetically defined syngeneic mouse models of ovarian cancer as tools for the discovery of combination immunotherapy”.* Abbreviations: mFTE – murine fallopian tube epithelium, OE – overexpression.

Validation of mFTE cell lines harboring patient-relevant mutant genotypes

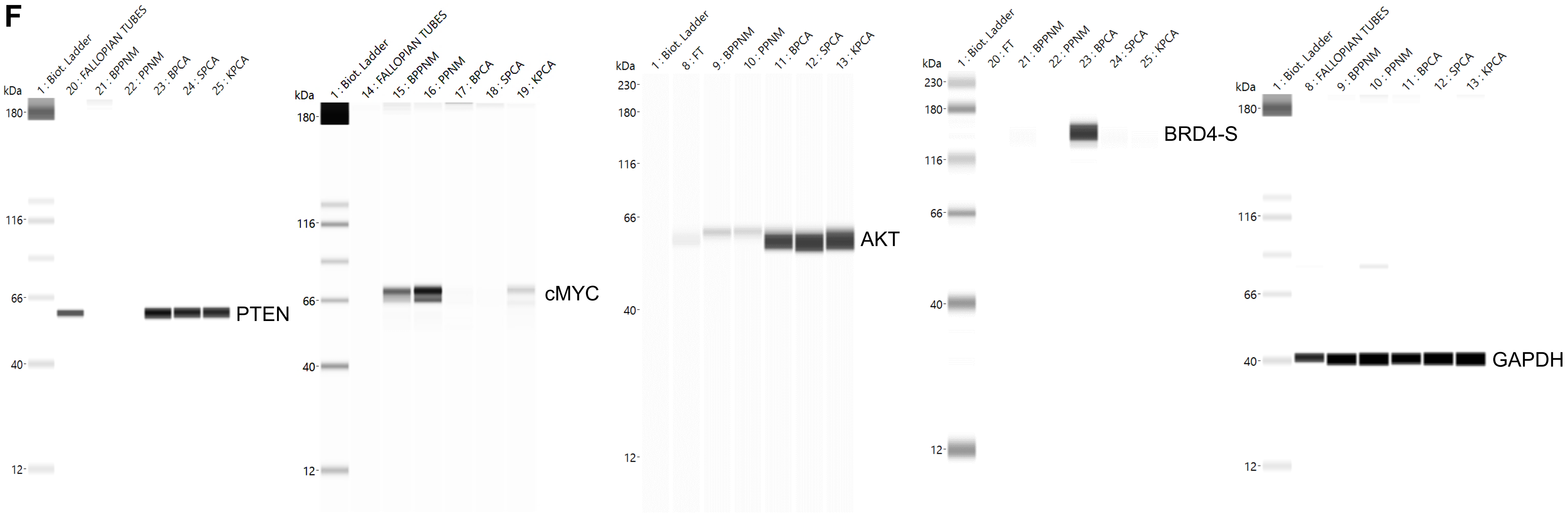

Supplementary Figure S1. (F) Uncropped WES images showing the expression of PTEN, cMYC, AKT, BRD4-S and GAPDH (loading control) in a panel of mFTE cell lines vs. normal mouse fallopian tubes.

SDHA levels in mFTE cell lines

G

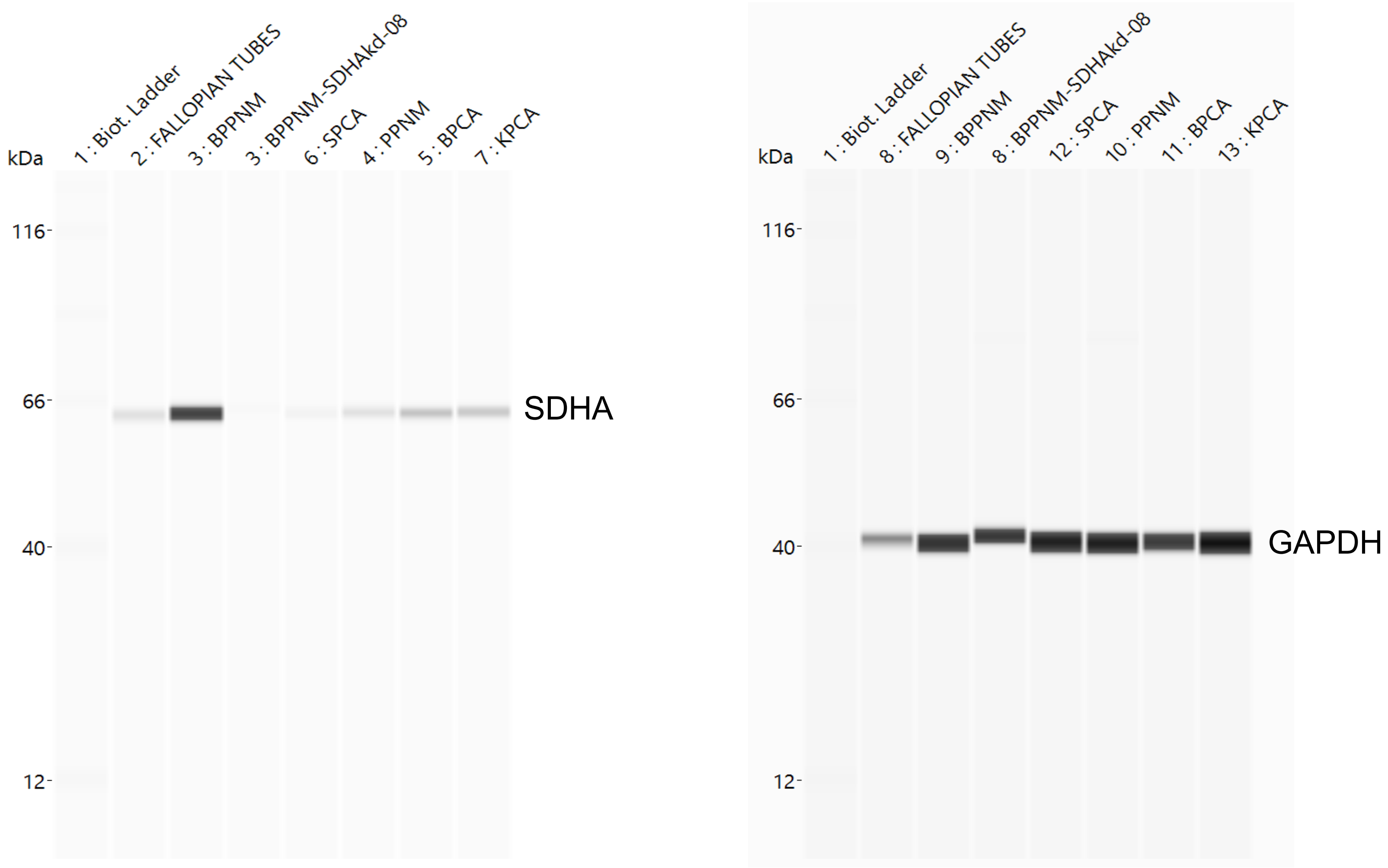

Supplementary Figure S1. (G) Uncropped WES images showing the expression of SDHA and GAPDH (loading control) in a panel of mFTE cell lines vs. normal mouse fallopian tubes.

SDHA levels in human ovarian cancer cell lines

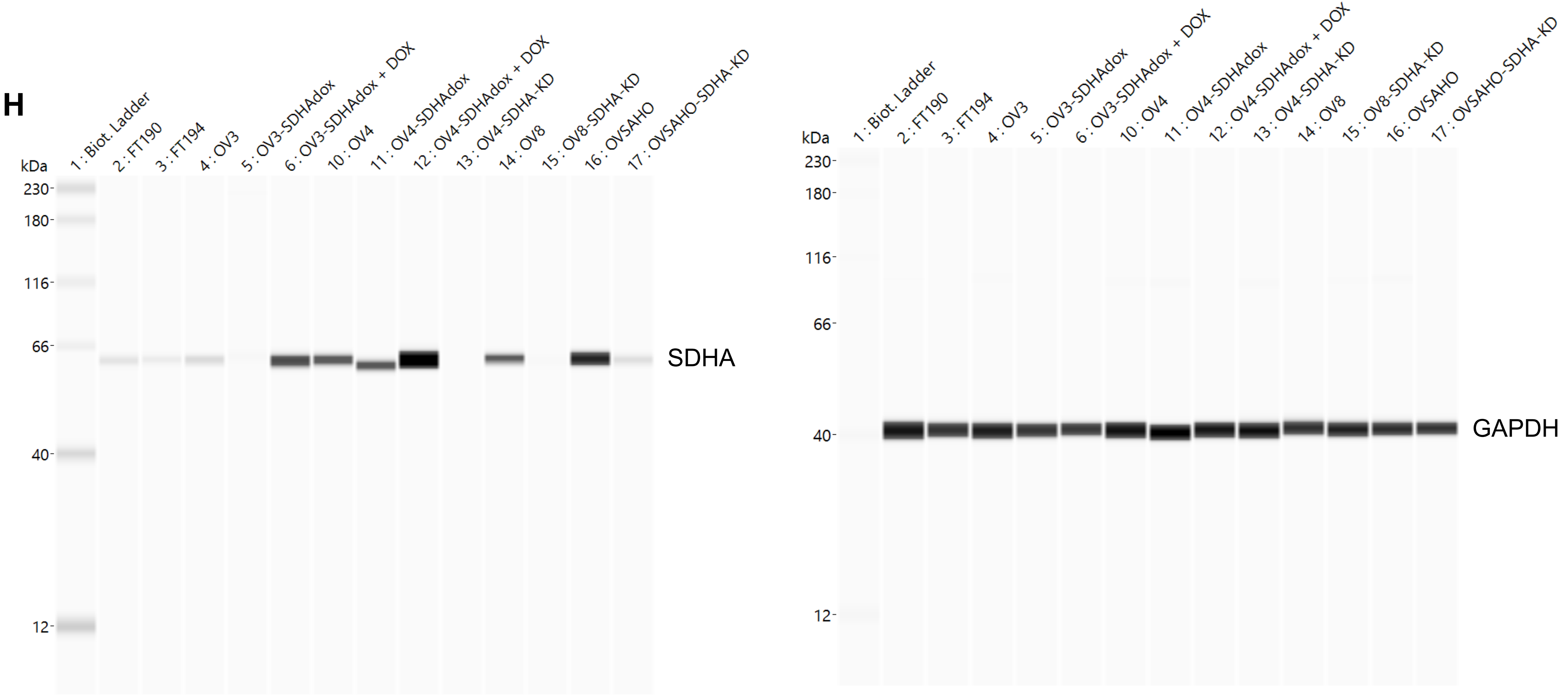

Supplementary Figure S1. (H) Uncropped WES images showing the expression of SDHA and GAPDH (loading control) in a panel of human ovarian cancer cell lines vs. normal human fallopian tube cell lines (FT190, FT194).
