# Supplementary material for: The Identification of a Key Regulator of Mitochondrial Metabolism, the LRPPRC Protein, as a Novel Therapeutic Target in SDHA-Overexpressing Ovarian Tumors
Source: Cancers (Basel). 2025 Jun 11;17(12):1942. doi: 10.3390/cancers17121942 (PMC12190274; doi:10.3390/cancers17121942)
Supplement: Supplementary file 1 [file cancers-17-01942-s001.zip › Supplementary Figure S2.pdf]

The impact of SDHA overexpression on human ovarian cancer cell proliferation in suspension cell cultures vs. adherent cell cultures

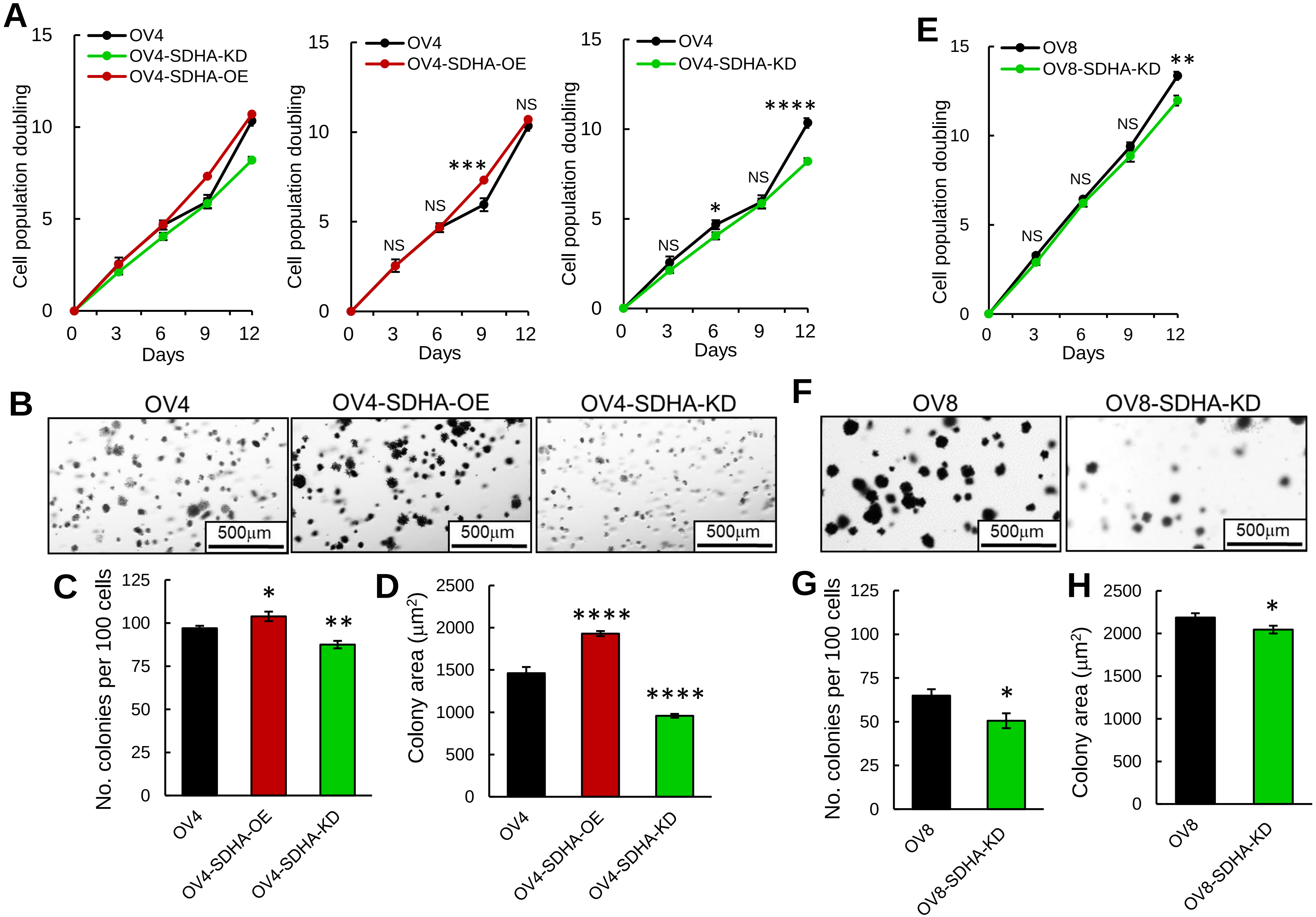

Supplementary Figure S2. (A) The effect of SDHA overexpression (OE) or knockdown (KD) on OVCAR4 (OV4) cell proliferation assessed by 3T5 cell doubling assay. In the right graph all growth curves are plotted together. In the middle and left graph, OV4-SDHA-OE or OV4-SDHA-KD growth curves are compared with parental OV4 control, respectively (unpaired t test). **(B)** Images represent anchorage-independent growth and colony formation of parental OVCAR4 cells compared with SDHA overexpressing or SDHA KD cell lines. Number **(C)** and size **(D)** of colonies were quantified and illustrated on graphs (one-way Anova). **(E)** The effect of SDHA KD on OVCAR8 (OV8) cell proliferation (3T5 cell doubling assay, unpaired t test). **(F)** Anchorage-independent growth and colony formation of parental OV8 cells compared with SDHA knockdown (OV8-SDHA-KD) cells. Number **(G)** and size **(H)** of colonies were quantified and illustrated on graphs (unpaired t test).

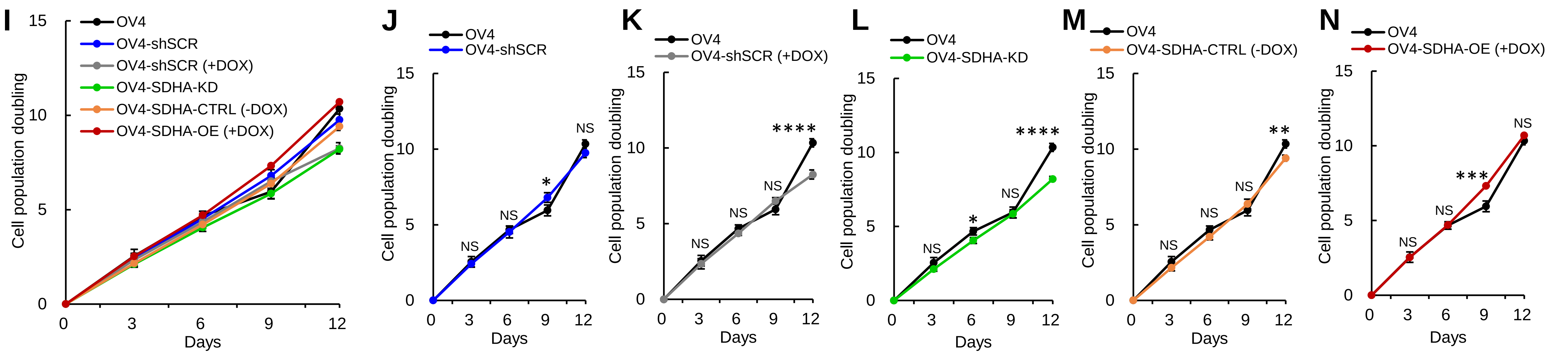

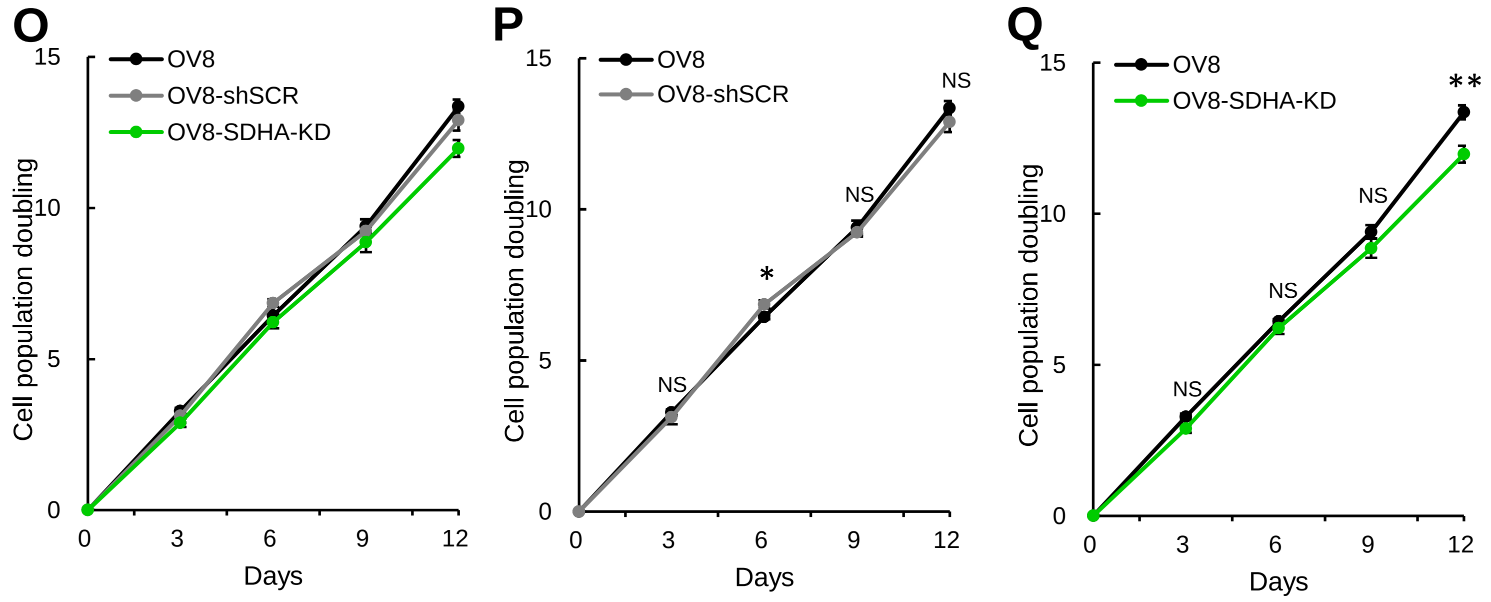

Supplementary Figure S2. (I) Cell proliferation of OVCAR4 (OV4) following dox inducible overexpression of SDHA (OV4-SDHA-OE +DOX) vs. respective controls or stable SDHA knockdown (OV4-SDHA-KD). Control cell lines included: OV4 cells overexpressing dox inducible shSCR (scrambled short hairpin) with or without dox induction (OV4-shSCR +DOX and OV4-shSCR, respectively), and cell line with SDHA overexpressing construct with and without dox stimulation (OV4-SDHA-OE +DOX and OV4-SDHA-CTRL –DOX, respectively). Cell proliferation was assessed by 3T5 cell doubling assay. (**J-N**). In individual graphs, each engineered cell line is compared with OV4 parental cells (unpaired t test). (**O**) Cell proliferation of OVCAR8 (OV8) following stable SDHA KD (OV8-SDHA-KD) vs. shSCR control cells (OV8-shSCR). (**P-Q**). In individual graphs, each engineered cell line is compared with OV8 parental cell line (unpaired t test).
