# Supplementary material for: The Identification of a Key Regulator of Mitochondrial Metabolism, the LRPPRC Protein, as a Novel Therapeutic Target in SDHA-Overexpressing Ovarian Tumors
Source: Cancers (Basel). 2025 Jun 11;17(12):1942. doi: 10.3390/cancers17121942 (PMC12190274; doi:10.3390/cancers17121942)
Supplement: Supplementary file 1 [file cancers-17-01942-s001.zip › Supplementary Figure S3.pdf]

Pilot study evaluating a survival of mice implanted orthotopically (into ovary or peritoneum) with tumor models endogenously overexpressing SDHA (BPPNM) or those with low SDHA levels (SPCA)

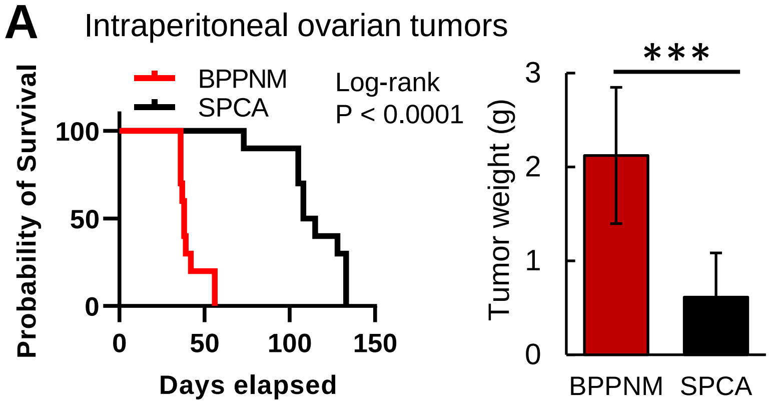

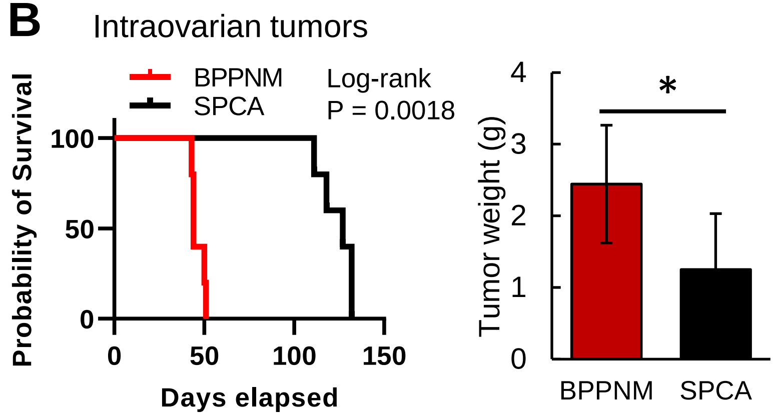

C

BPPNM (intraovarian tumors)					
Mouse number	M1	M2	M3	M4	M5
Tumor weight (g)	2.2	2.5	1.6	2.1	3.8
Resected tumor					
**SPCA (intraovarian tumors)**					
Mouse number	M1	M2	M3	M4	M5
Tumor weight (g)	1.1	2.6	1.0	0.6	0.9
Resected tumor					

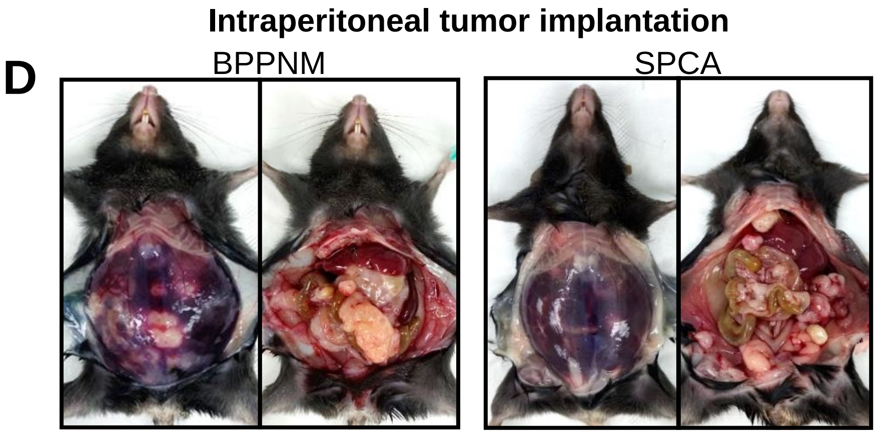

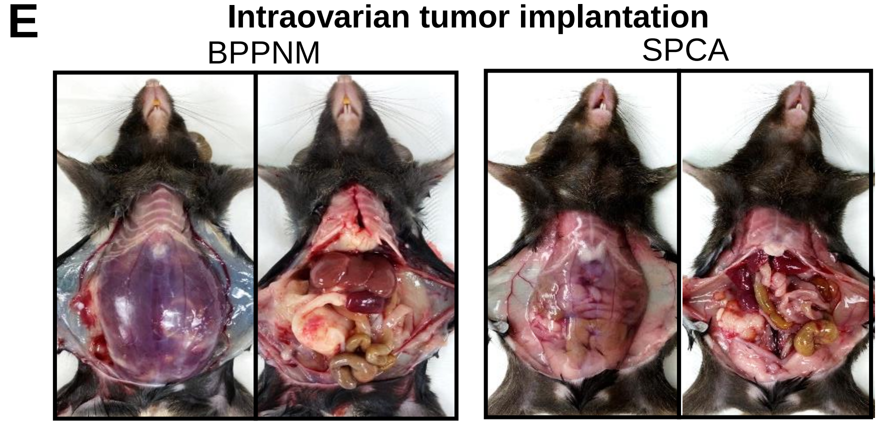

Supplementary Figure S3. (A) Kaplan-Meier curves showing % probability of survival of C57BL/6J female mice injected into peritoneum (IP) with $3x10^6$ SDHA overexpressing BPPNM or SDHA-low SPCA tumor cells. When mice reached advanced tumor burden and became moribund, the animals were euthanized and evaluated for survival time and tumor mass. The SDHA-high BPPNM tumor model showed significantly increased tumorigenic potential when compared with SDHA-low tumors (SPCA), resulting in increased tumor mass (unpaired t test) and substantially reduced mouse survival (Kaplan Meier method and Log-Rank Mantel-Cox test). **(B)** Survival of C57BL/6J female mice injected into ovary with $0.5x10^6$ BPPNM or SPCA tumor cells. The BPPNM tumors showed increased tumor mass (unpaired t test) and significantly reduced mouse survival (Kaplan Meier method and Log-Rank Mantel-Cox test) when compared with SPCA tumors. (**C)** Intraovarian tumors harvested from mice described in 'B'. BPPNM tumors are larger than SPCA counterparts, which is calculated in the graph shown in 'B'. **(D-E)** Representative images of ascites and metastases in C57BL/6J female mice implanted with BPPNM or SPCA tumor cells IP **(D)** or into ovary **(E)**.

Survival of mice implanted orthotopically (into ovary) with tumor models endogenously overexpressing SDHA (BPPNM), those with naturally low SDHA levels (SPCA), or SDHA knockdown (BPPNM-SDHA-KD)

F

BPPNM									
**Mouse number**	M1	M2	M3	M4	M5	M6	M7	M8	M9
**Tumor weight (g)**	4.4	3.3	3.8	1.9	3.5	3.9	3.6	3.3	2.4
**Resected tumor**	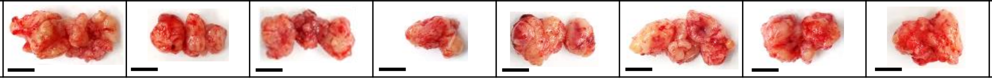								

BPPNM-SDHA-KD									
**Mouse number**	M1	M2	M3	M5	M6	M7	M8	M9	M10
**Tumor weight (g)**	4.5	3.8	2.6	3.5	4.8	2.7	3.7	3.9	3.3
**Resected tumor**	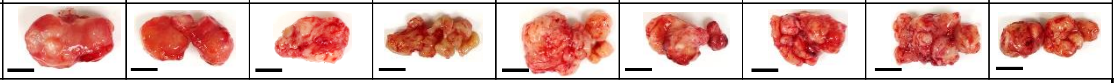								

SPCA									
**Mouse number**	M1	M2	M3	M4	M5	M7	M13	M29	
**Tumor weight (g)**	1.1	2.5	2.8	0.9	3.9	1.8	2.9	1.4	
**Resected tumor**	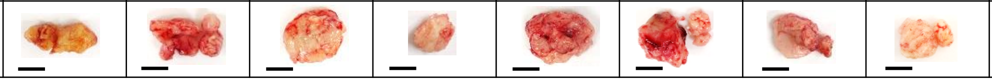								

G

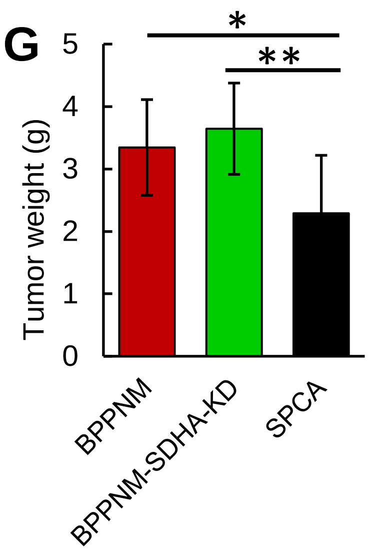

H

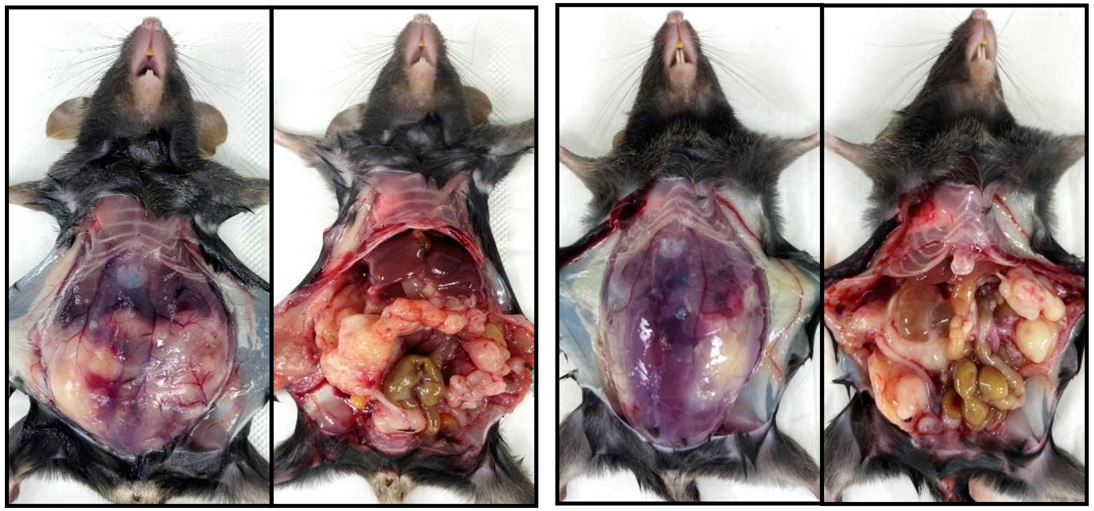

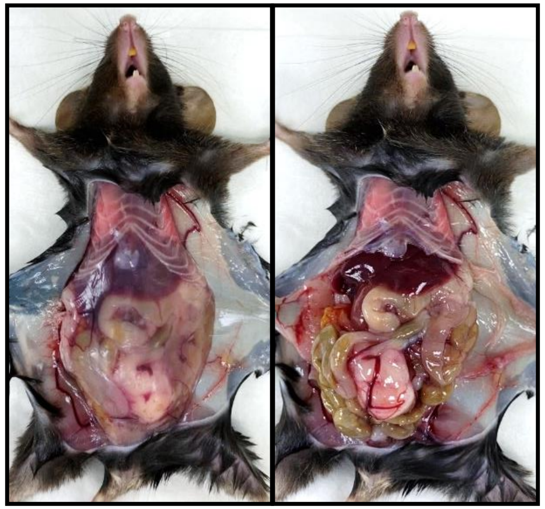

Supplementary Figure S3. (F) Intraovarian tumors with and without SDHA overexpression harvested from C57BL/6J female mice after reaching advanced tumor burden. Mice were injected into ovary with 0.5x10^6 tumor cells and monitored for tumor growth and survival (shown in Figure 1H). **(G)** BPPNM +/- SDHA-KD tumors are larger than SPCA counterparts, which is calculated in the graph (one-way Anova). Asterisks indicate level of statistical significance: * P ≤ 0.1, **P ≤ 0.01. **(H)** Macroscopic evaluation of animals revealed that mice implanted with BPPNM or BPPNM-SDHA-KD tumor cells demonstrated more robust metastasis and ascites than mice implanted with SPCA tumor cells.
