# Supplementary material for: The Identification of a Key Regulator of Mitochondrial Metabolism, the LRPPRC Protein, as a Novel Therapeutic Target in SDHA-Overexpressing Ovarian Tumors
Source: Cancers (Basel). 2025 Jun 11;17(12):1942. doi: 10.3390/cancers17121942 (PMC12190274; doi:10.3390/cancers17121942)
Supplement: Supplementary file 1 [file cancers-17-01942-s001.zip › Supplementary Figure S5.pdf]

Effects of SDHA overexpression in ovarian cancer on glucose and glutamine flux

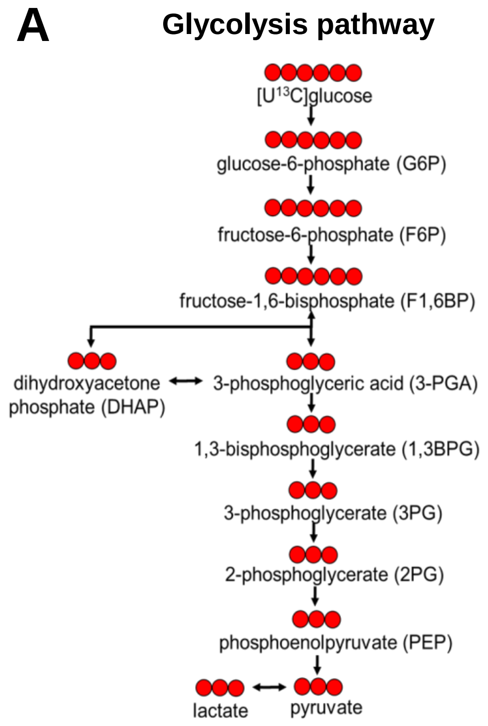

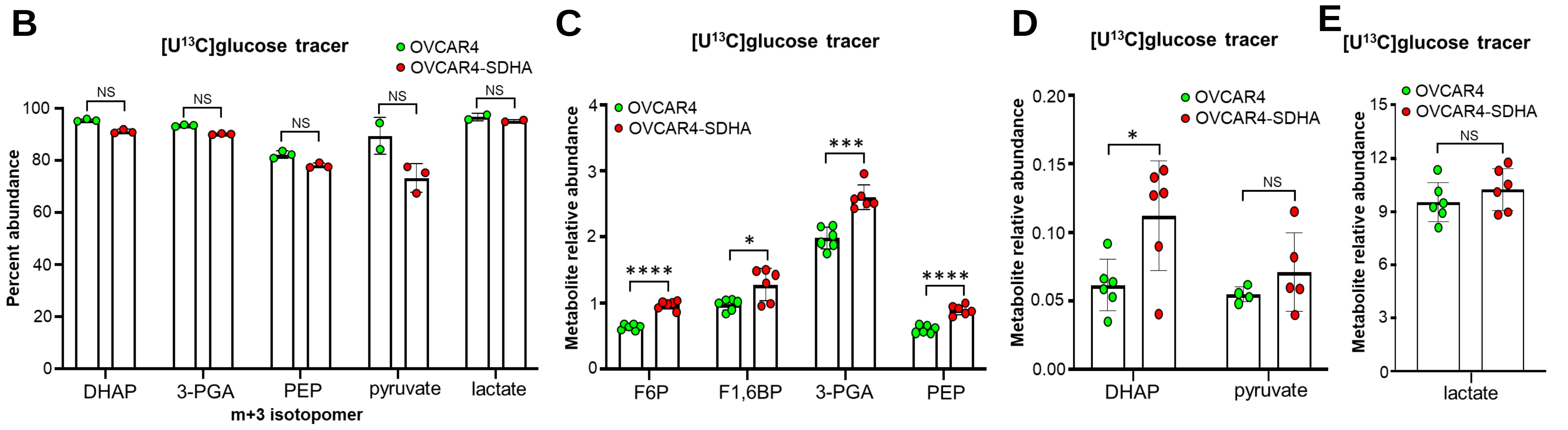

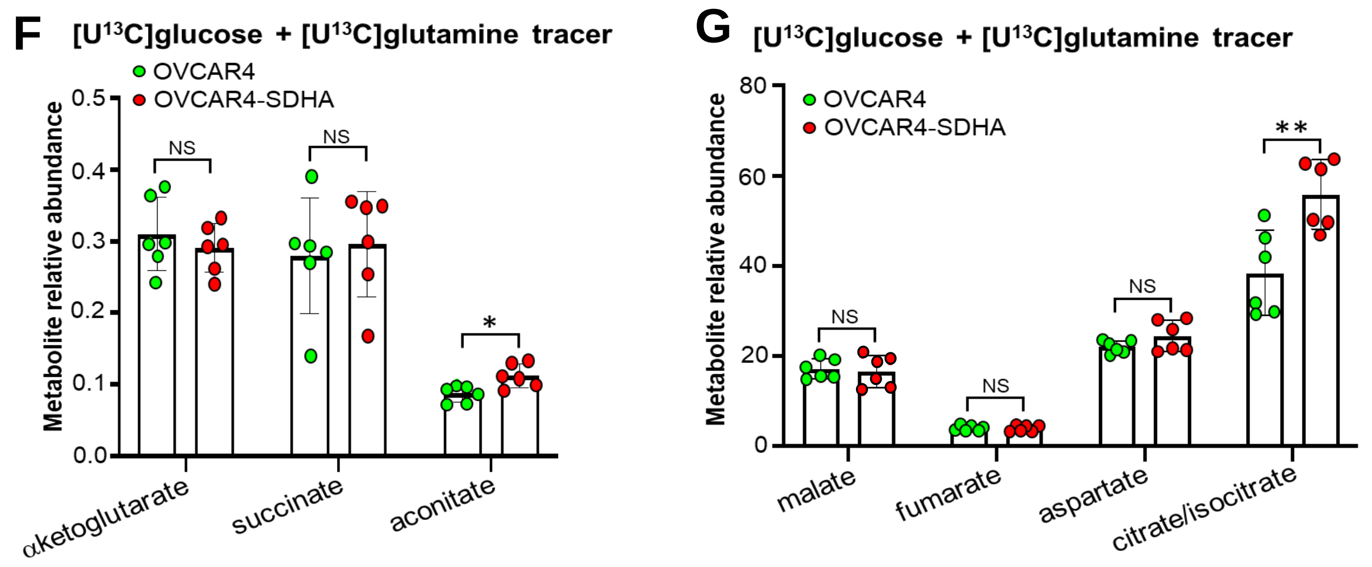

Supplementary Figure S5. (A) The diagram represents the flux of metabolic intermediates derived from a single tracer [U^{13}C]glucose via glycolysis pathway in OVCAR4 cell lines. Red circles indicate ^{13}C. **(B)** Graph represent the percentage abundance of the m+3 isotopomers within the glycolysis metabolite pool in OVCAR4-SDHA vs. OVCAR4 control cells following tracing of [U^{13}C]glucose (unpaired t test). **(C, D, E)** Graphs show differences in relative abundance of glycolysis pathway metabolites between OVCAR4-SDHA vs. OVCAR4 cells following tracing of [U^{13}C]glucose (unpaired t test). **(F-G)** Differences in relative abundance of TCA cycle pathway metabolites between OVCAR4-SDHA vs. OVCAR4 cells following parallel tracing of [U^{13}C]glucose and [U^{13}C]glutamine (unpaired t test). **(B-G)** Asterisks indicate level of statistical significance: * P ≤ 0.05, ** P ≤ 0.01, ***P ≤ 0.001, ****P ≤ 0.001, NS not significant.

Effects of SDHA overexpression in ovarian cancer on glucose and glutamine flux

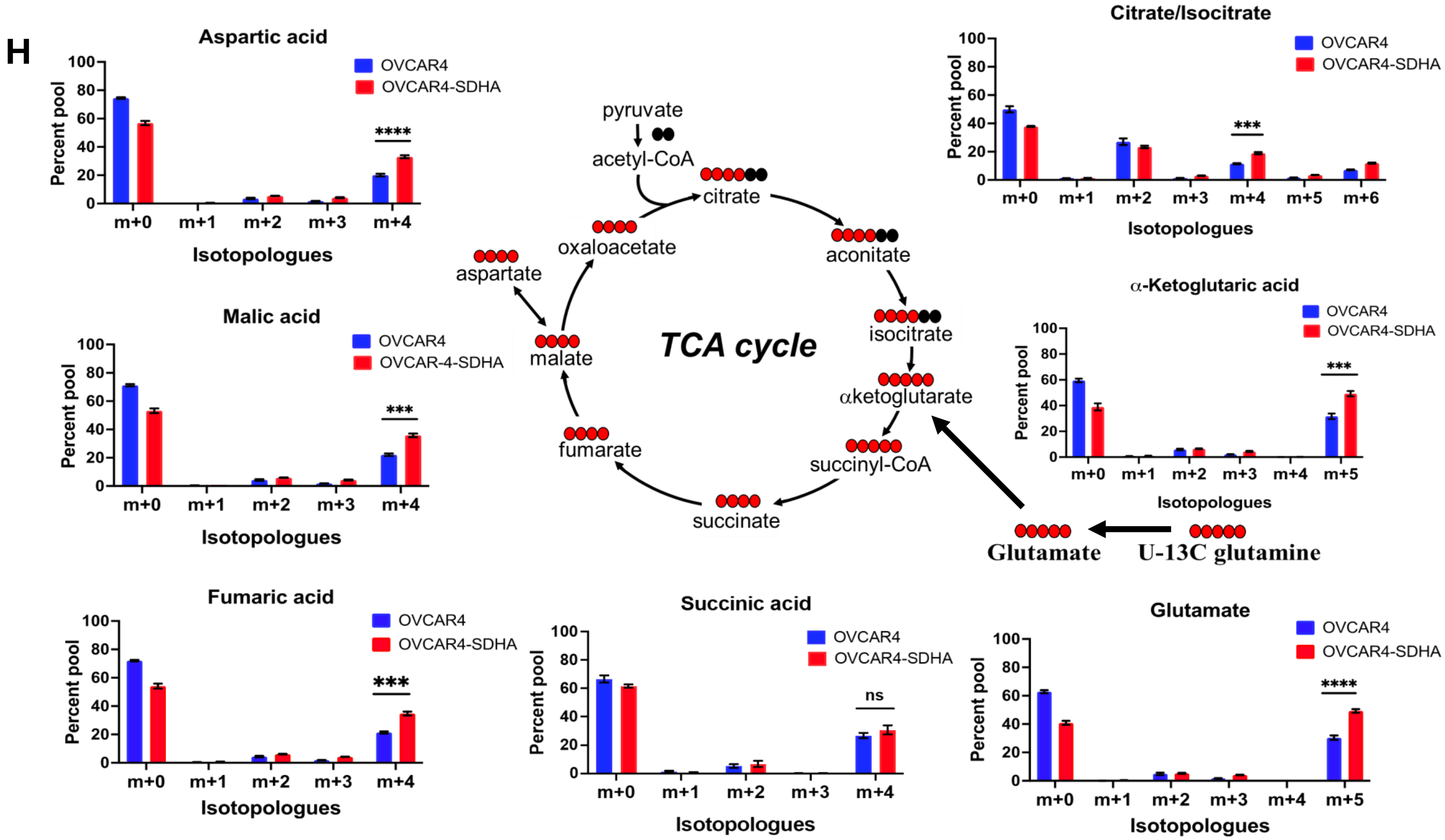

Supplementary Figure S5. (H) The diagram shows incorporation of ^{13}C stable isotopes into the TCA cycle intermediates in OVCAR4-SDHA cells vs. control OVCAR4 cells following parallel tracing of [U^{13}C]glucose and [U^{13}C]glutamine. Black circles indicate ^{13}C carbon contribution from [U^{13}C]glucose, while red circles indicate ^{13}C carbon contribution from [U^{13}C]glutamine. Statistical analysis was performed using unpaired t test. Asterisks indicate level of statistical significance: ***P ≤ 0.001, ****P ≤ 0.001, NS not significant.

Effects of SDHA overexpression in ovarian cancer on mitochondrial respiration and ATP generation

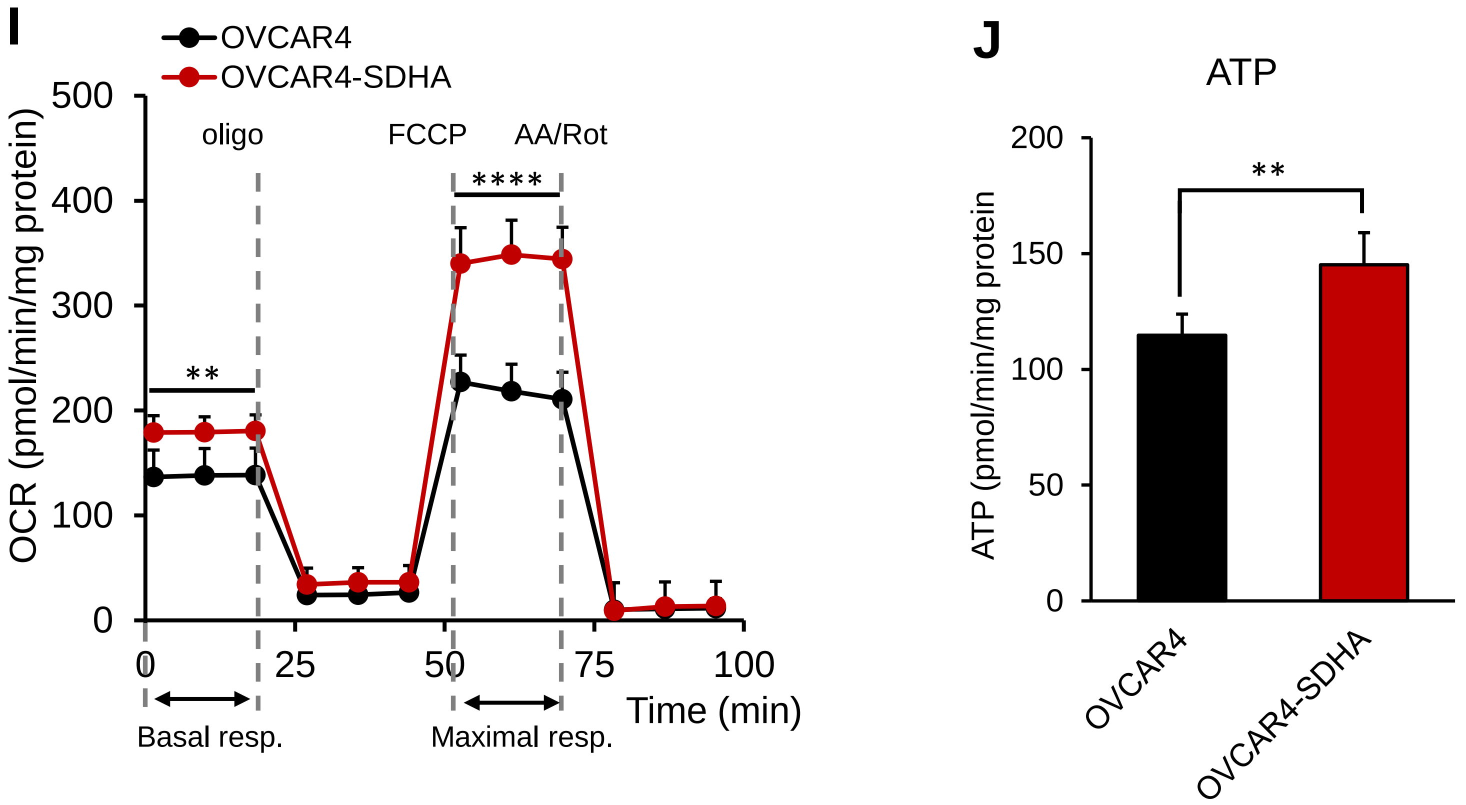

Supplementary Figure S5. (I) Oxygen consumption rate (OCR) was measured in OVCAR4 cell lines with and without SDHA overexpression by the Seahorse XF Cell Mito Stress Test. Cells were challenged by oligomycin A "oligo", FCCP, and antimycin A + rotenone "AA/Rot". Basal OCR (basal respiration) represents the mitochondrial respiration at resting state; the maximal respiration indicates the cellular response to an increased ATP demand during periods of high-energy expenditure. **(J)** Total ATP production rate in OVCAR4-SDHA and OVCAR4 control (Seahorse). **(I-J)** Statistical analysis was performed using unpaired t test. Asterisks indicate level of statistical significance: **P ≤ 0.01, ****P ≤ 0.001.
