# Supplementary material for: The Identification of a Key Regulator of Mitochondrial Metabolism, the LRPPRC Protein, as a Novel Therapeutic Target in SDHA-Overexpressing Ovarian Tumors
Source: Cancers (Basel). 2025 Jun 11;17(12):1942. doi: 10.3390/cancers17121942 (PMC12190274; doi:10.3390/cancers17121942)
Supplement: Supplementary file 1 [file cancers-17-01942-s001.zip › Supplementary Figure S7.pdf]

Analysis of a direct binding of shikonin to LRPPRC protein assessed by pull-down assay

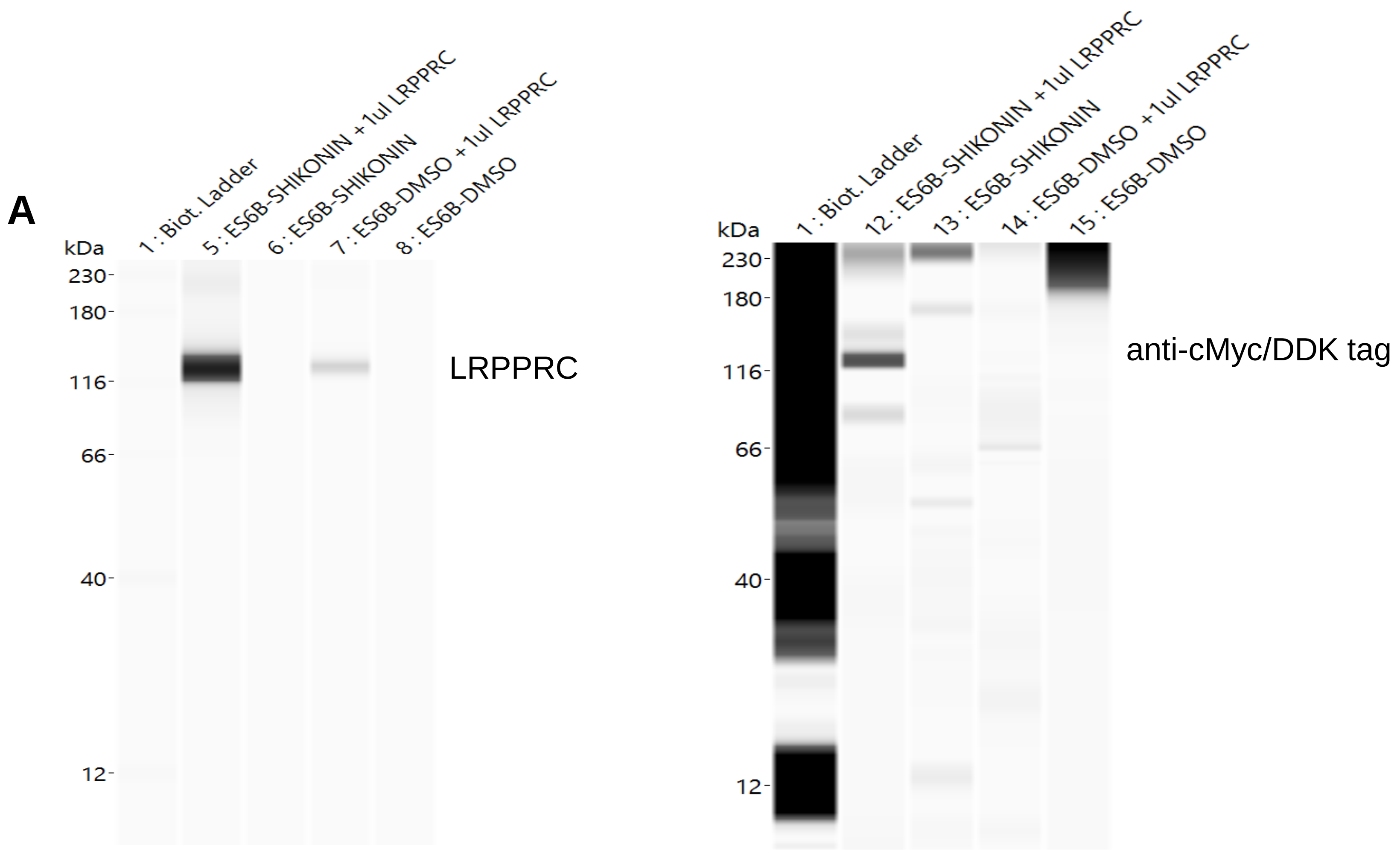

Supplementary Figure S7. (A) Uncropped WES images (shown in Figure 4B in main manuscript) detecting the expression of cMyc/DDK-tagged recombinant LRPPRC protein bound by shikonin. Shikonin binds with high affinity to LRPPRC protein as shown by pull-down assay using shikonin-conjugated epoxy-activated Sepharose-6B (ES6B) and OVCAR4 cell extracts +/- recombinant LRPPRC probed with antibodies against LRPPRC or cMyc/DDK tag.

SDHA and LRPPRC levels in mFTE cell lines

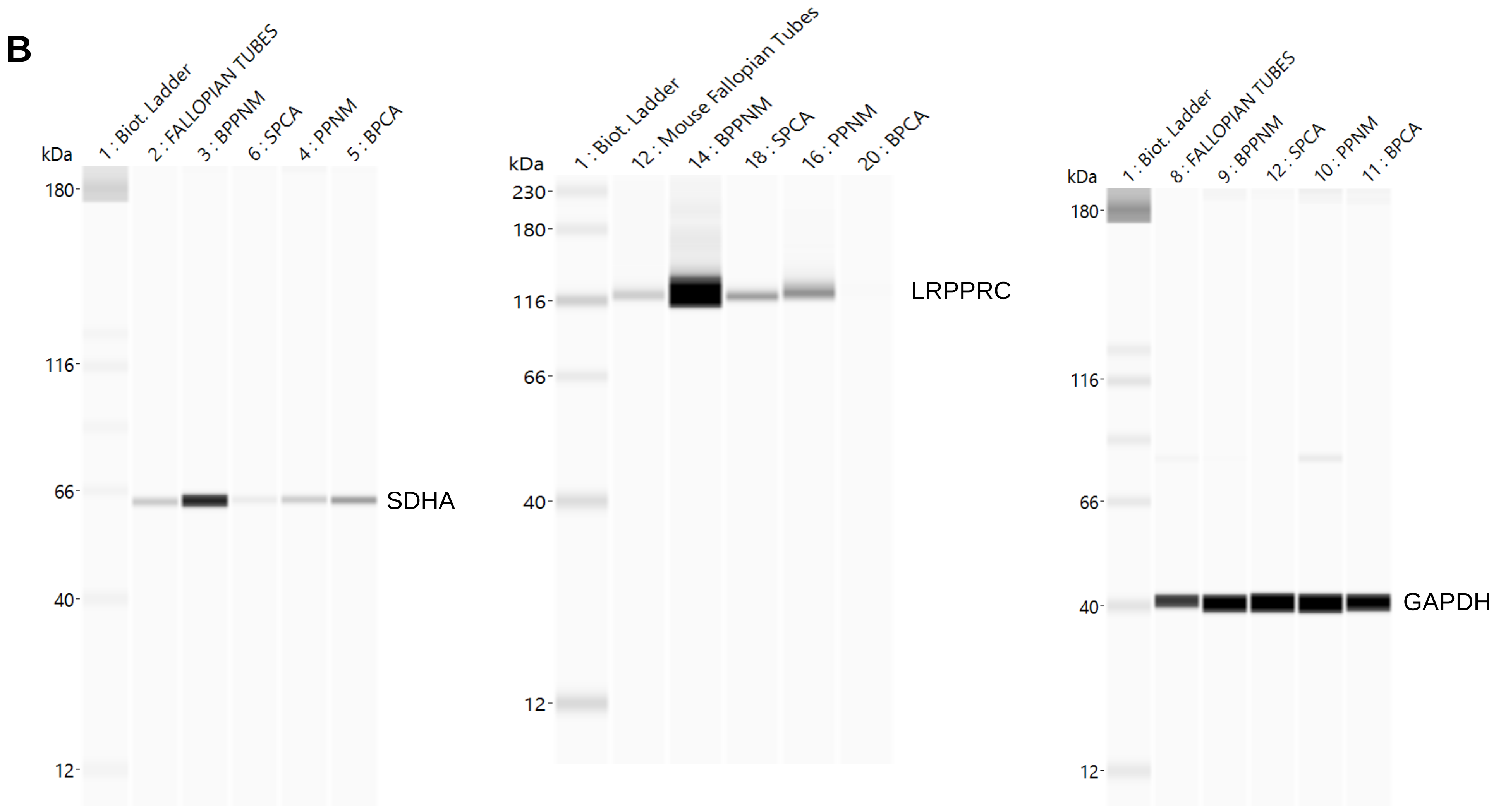

Supplementary Figure S7. (B) Uncropped WES images (shown in Figure 4C in main manuscript) showing the expression of SDHA, LRPPRC and GAPDH (loading control) in a panel of mFTE cell lines vs. normal mouse fallopian tubes.

SDHA and LRPPRC levels in human ovarian cancer cell lines

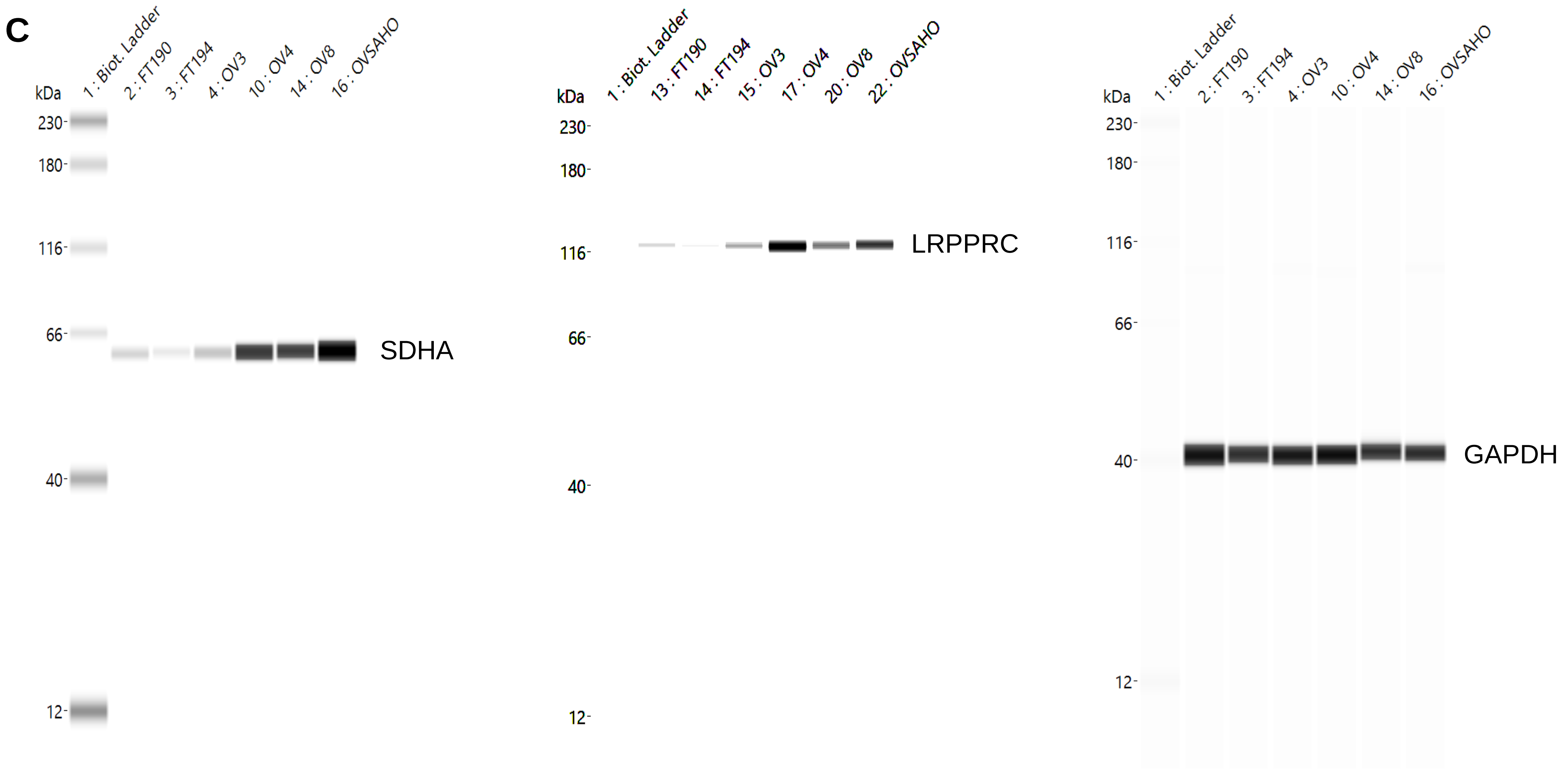

Supplementary Figure S7. (C) Uncropped WES images (shown in Figure 4D in main manuscript) showing the expression of SDHA, LRPPRC and GAPDH (loading control) in a panel of human ovarian cancer cell lines vs. normal human fallopian tube cell lines (FT190, FT194).

SDHA and LRPPRC levels in HGSOC PDXs

E

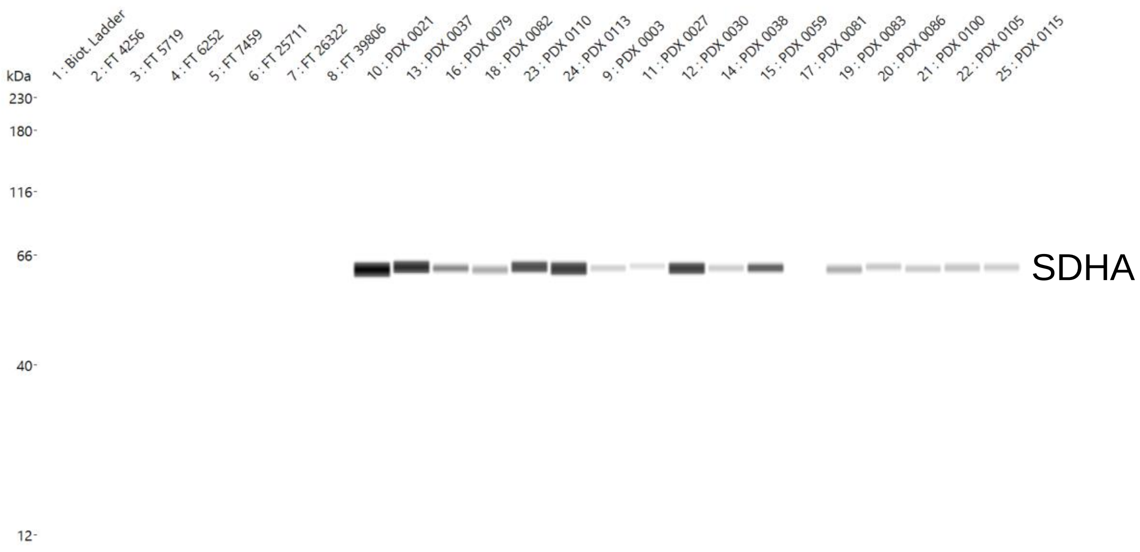

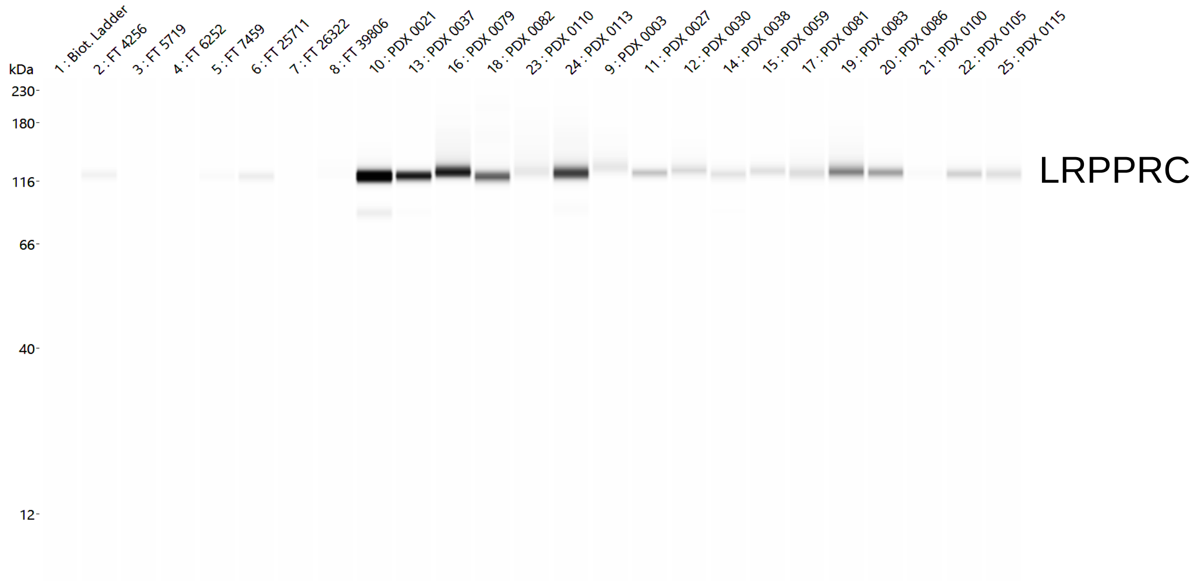

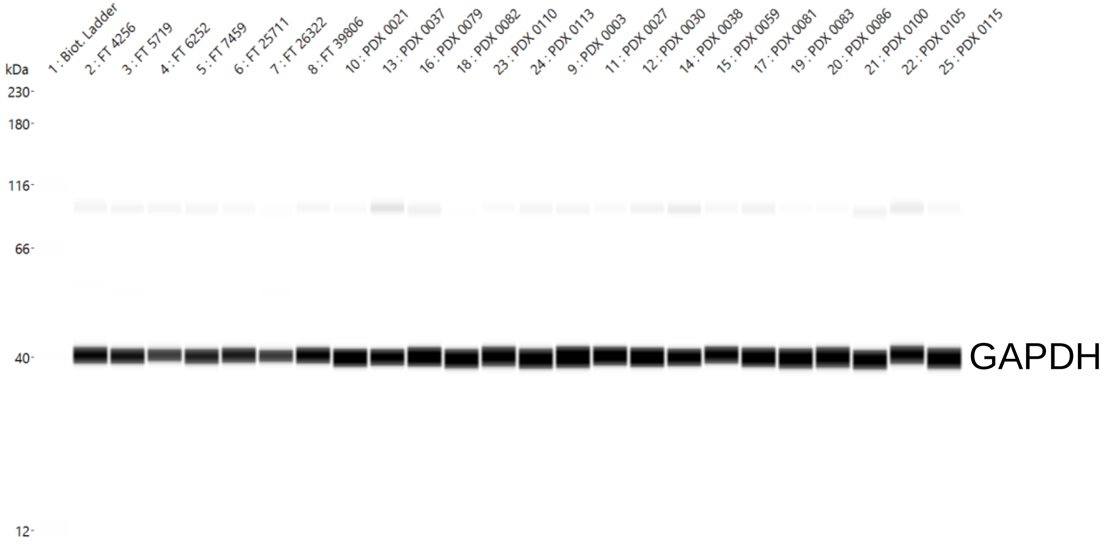

Supplementary Figure S7. (E) Uncropped WES images (shown in Figure 4E in main manuscript) showing the expression of SDHA, LRPPRC and GAPDH (loading control) in a panel of high grade serous ovarian cancer (HGSOC) PDX models 'PDX' vs. normal human fallopian tube samples from ovarian cancer patients 'FT'.

SDHA and LRPPRC levels in mFTE cell lines following LRPPRC-KD

F

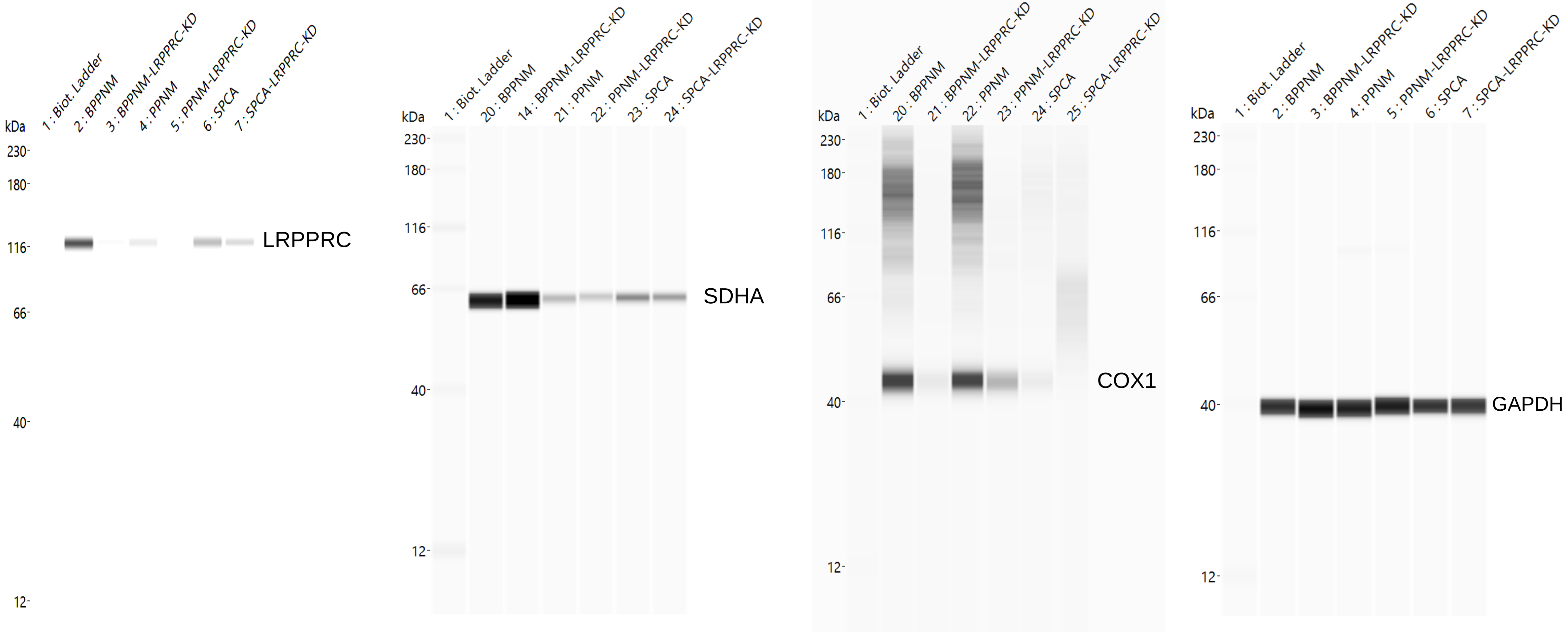

Supplementary Figure S7. (F) Uncropped WES images (shown in Figure 4G in main manuscript) showing the expression of SDHA, LRPPRC, COX1, and GAPDH (loading control) in a panel of mFTE cell lines with and without LRPPRC-KD.

SDHA and LRPPRC levels in human ovarian cancer cell lines following LRPPRC-KD

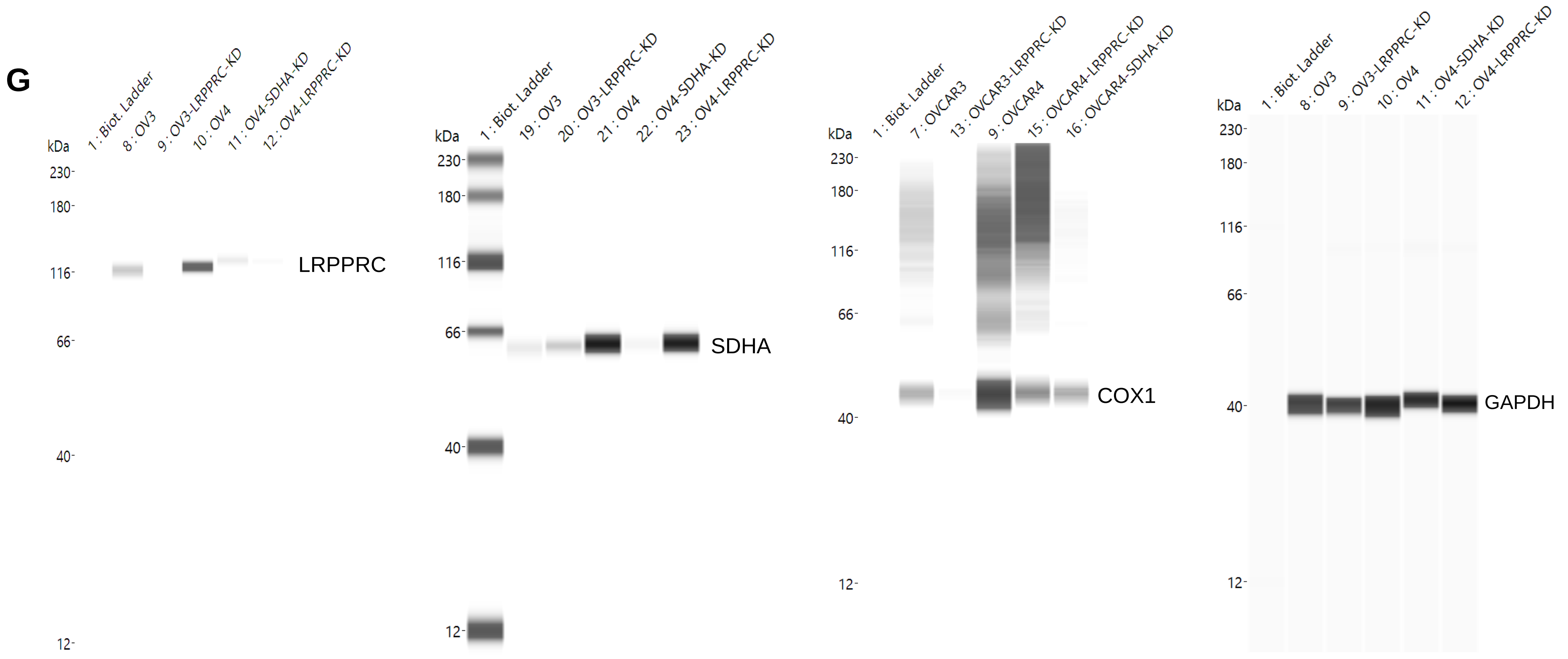

Supplementary Figure S7. (G) Uncropped WES images (shown in Figure 4H in main manuscript) showing the expression of SDHA, LRPPRC, COX1, and GAPDH (loading control) in a panel of human ovarian cancer cell lines with and without SDHA-KD or LRPPRC-KD.

LRPPRC loss and apoptosis in mFTE ovarian cancer cell lines following treatment with shikonin

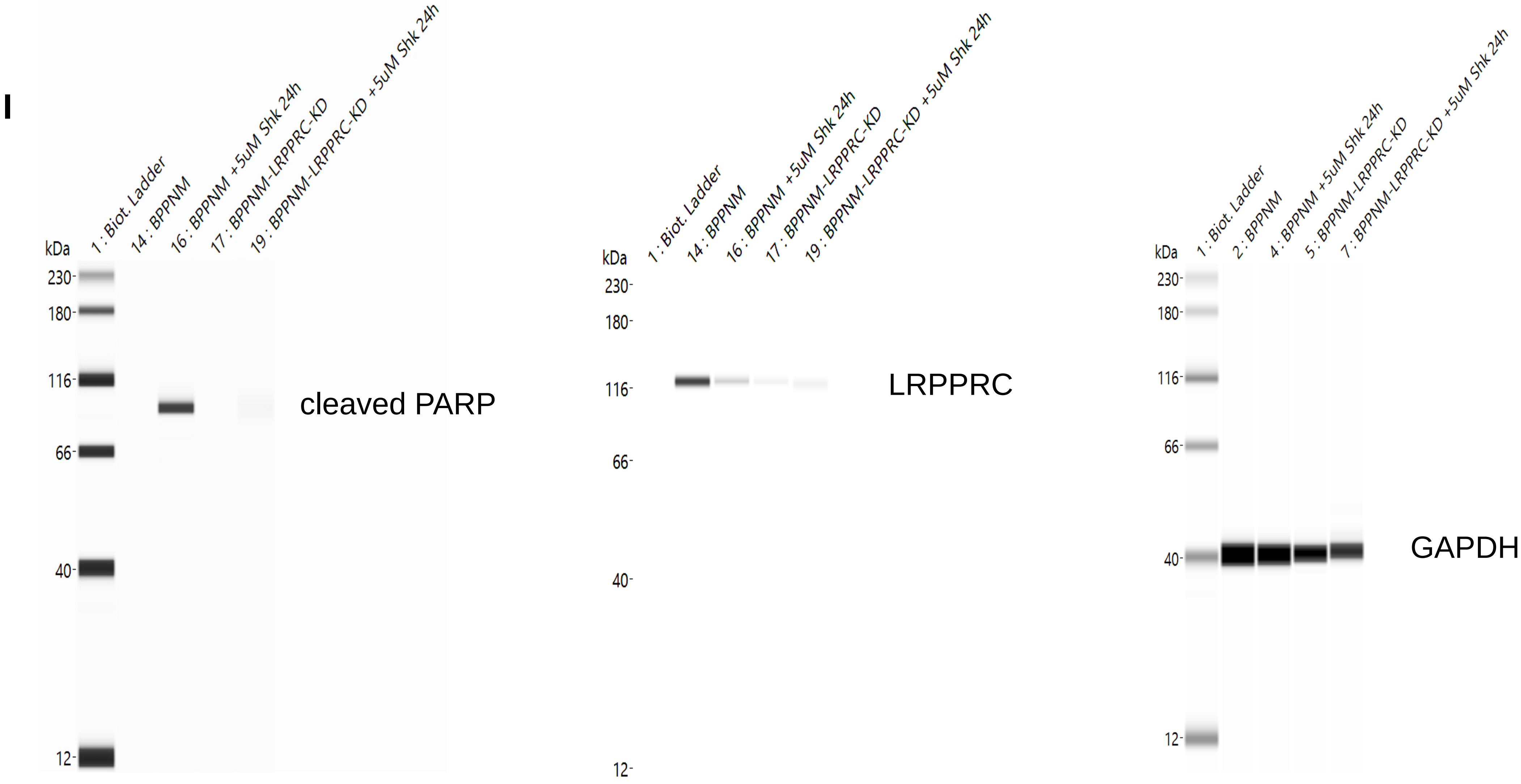

Supplementary Figure S7. (I) Uncropped WES images (shown in Figure 4I in main manuscript) showing the expression of cleaved PARP (apoptosis marker), LRPPRC and GAPDH (loading control) in a panel of mFTE cell lines treated with 5 μM dose of shikonin for 24 h *in vitro*. Shikonin treatment leads to a loss of LRPPRC expression and triggers apoptosis.

LRPPRC loss and apoptosis in human ovarian cancer cell lines following treatment with shikonin

J

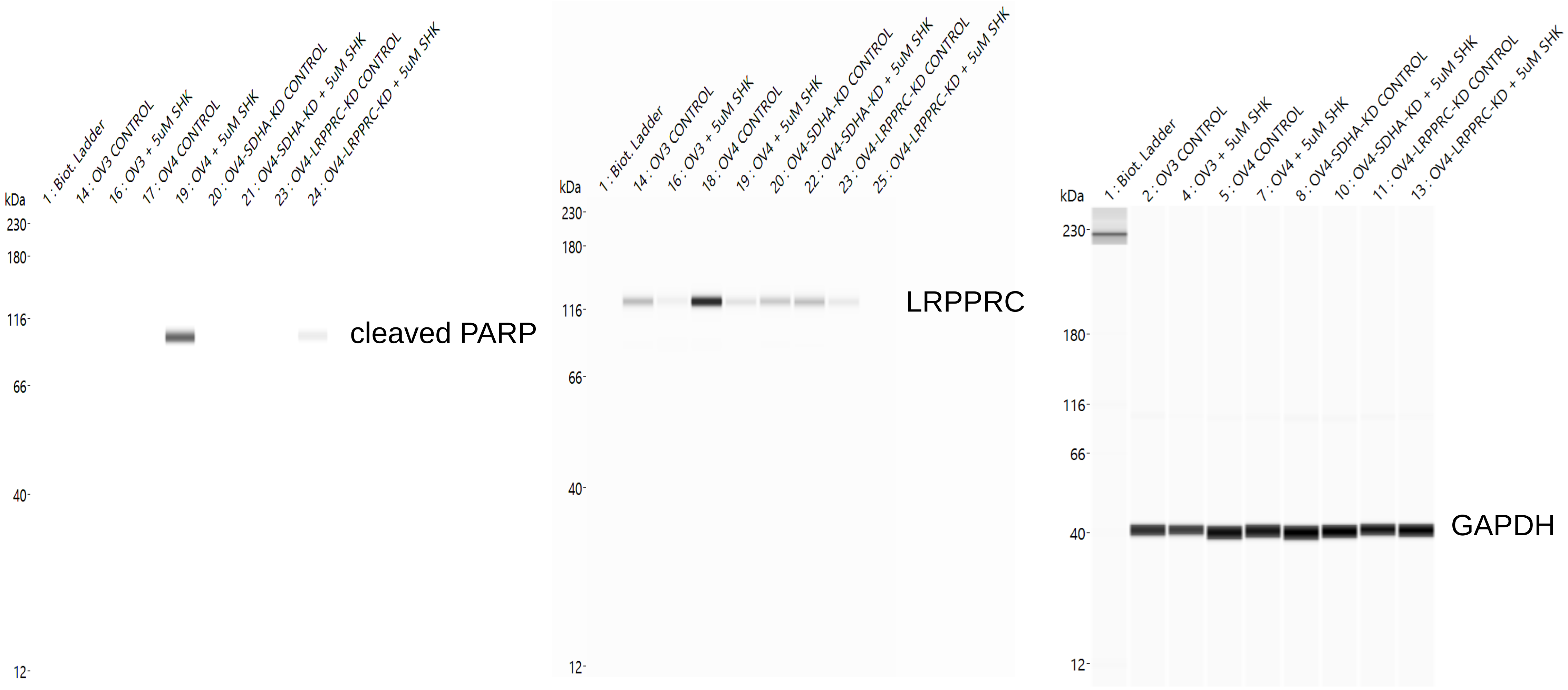

Supplementary Figure S7. (J) Uncropped WES images (shown in Figure 4J in main manuscript) showing the expression of cleaved PARP (apoptosis marker), LRPPRC and GAPDH (loading control) in a panel of human ovarian cancer cell lines treated with 5 μM dose of shikonin for 24 h *in vitro*. Shikonin treatment leads to a loss of LRPPRC expression and triggers apoptosis.
