# Supplementary material for: The Identification of a Key Regulator of Mitochondrial Metabolism, the LRPPRC Protein, as a Novel Therapeutic Target in SDHA-Overexpressing Ovarian Tumors
Source: Cancers (Basel). 2025 Jun 11;17(12):1942. doi: 10.3390/cancers17121942 (PMC12190274; doi:10.3390/cancers17121942)

SDHA and *LRPPRC* gene expression during HGSOC development and progression

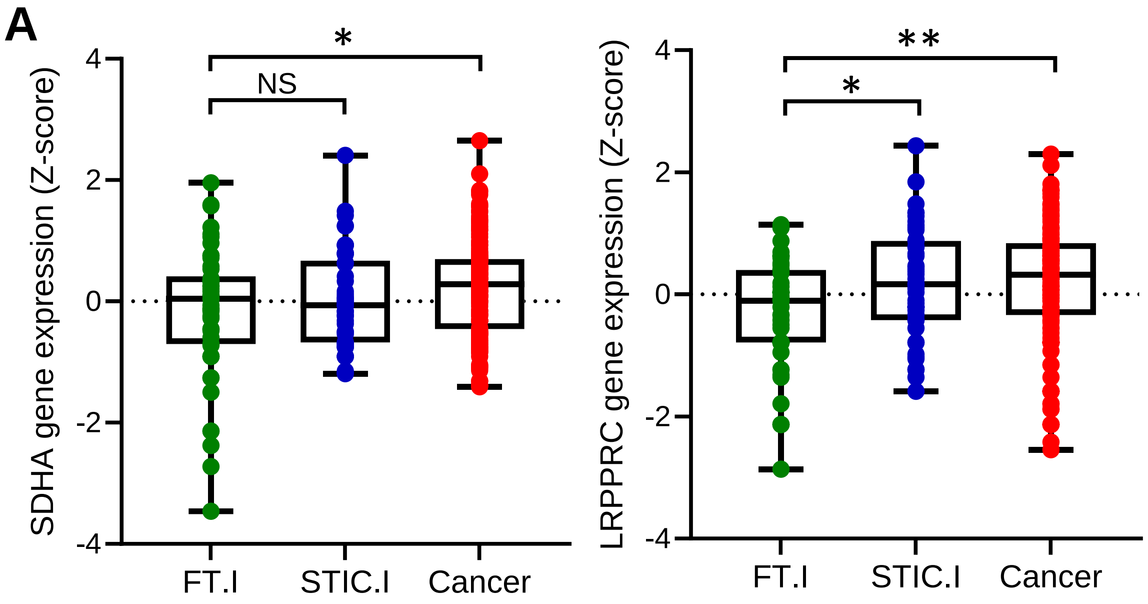

Supplementary Figure S8. *SDHA* and *LRPPRC* gene expression during HGSOC development and progression. (A) Box plots showing the comparison of *SDHA* gene expression (Z-score normalized data) in human normal epithelium of incidental FT (FT.I), incidental STIC lesions (STIC.I) and advanced cancer. The positive Z-scores indicates upregulation, whereas negative scores indicate down-regulation. The *SDHA* and *LRPPRC* expression increases as FT.I progresses to malignant cancer reaching significantly higher expression levels in advanced HGSOC (one-way ANOVA). Asterisks indicate level of statistical significance: * P ≤ 0.05, ** P ≤ 0.01. **(B)** Assessment of correlation between *SDHA* and *LRPPRC* gene expression across normal human FT, precancerous lesions (p53 signature, STIC lesions) and invasive cancer. Calculation of Pearson correlation coefficient (r) revealed statistically significant positive correlation between *SDHA* and *LRPPRC* expression in STIC and cancer, with a particularly strong correlation in the invasive cancer.

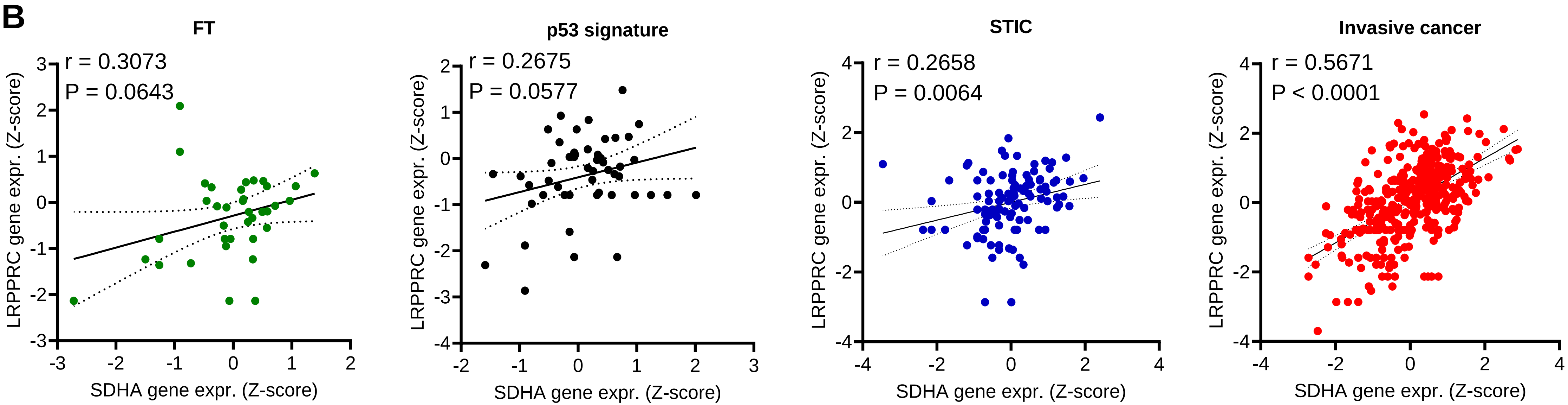

Supplement: Supplementary file 1 [file cancers-17-01942-s001.zip › Supplementary Figure S8.pdf]
